# Medium-chain fatty acids modulate myocardial function via a cardiac odorant receptor

**DOI:** 10.1007/s00395-017-0600-y

**Published:** 2017-01-23

**Authors:** Nikolina Jovancevic, A. Dendorfer, M. Matzkies, M. Kovarova, J. C. Heckmann, M. Osterloh, M. Boehm, L. Weber, F. Nguemo, J. Semmler, J. Hescheler, H. Milting, E. Schleicher, L. Gelis, H. Hatt

**Affiliations:** 1 Department of Cell Physiology, Ruhr-University Bochum, 44801 Bochum, Germany; 2 Walter Brendel Centre of Experimental Medicine, Ludwig-Maximilians-University, 80336 Munich, Germany; 3 Institute for Neurophysiology, University of Cologne, 50931 Cologne, Germany; 4 Erich and Hanna Klessmann Institute, Clinic for Thoracic and Cardiovascular Surgery, Heart and Diabetes Center NRW, Ruhr-University Bochum, 32545 Bad Oeynhausen, Germany; 5 Division of Pathobiochemistry and Clinical Chemistry, University of Tuebingen, 72076 Tuebingen, Germany; 6 DZHK (German Center for Cardiovascular Research), Partner Site Munich Heart Alliance, Munich, Germany

**Keywords:** Olfactory receptor, Calcium transients, Negative chronotropic effect, Negative inotropic effect, Medium-chain fatty acids

## Abstract

Several studies have demonstrated the expression of odorant receptors (OR) in various human tissues and their involvement in different physiological and pathophysiological processes. However, the functional role of ORs in the human heart is still unclear. Here, we firstly report the functional characterization of an OR in the human heart. Initial next-generation sequencing analysis revealed the OR expression pattern in the adult and fetal human heart and identified the fatty acid-sensing OR51E1 as the most highly expressed OR in both cardiac development stages. An extensive characterization of the OR51E1 ligand profile by luciferase reporter gene activation assay identified 2-ethylhexanoic acid as a receptor antagonist and various structurally related fatty acids as novel OR51E1 ligands, some of which were detected at receptor-activating concentrations in plasma and epicardial adipose tissue. Functional investigation of the endogenous receptor was carried out by Ca^2+^ imaging of human stem cell-derived cardiomyocytes. Application of OR51E1 ligands induced negative chronotropic effects that depended on activation of the OR. OR51E1 activation also provoked a negative inotropic action in cardiac trabeculae and slice preparations of human explanted ventricles. These findings indicate that OR51E1 may play a role as metabolic regulator of cardiac function.

**Electronic supplementary material:**

The online version of this article (doi:10.1007/s00395-017-0600-y) contains supplementary material, which is available to authorized users.

## Introduction

Transmembrane signal transduction by membrane receptor proteins enables a cell to convert an extracellular signal into cellular responses. Many important cell recognition and communication processes are mediated by a superfamily of transmembrane proteins, the G protein-coupled receptors (GPCRs). GPCRs play a prominent role in sensing extracellular signals, which cover a broad spectrum from physical to chemical stimuli, e.g., transmitters, hormones or ligands for taste and smell. The significance of these receptors is reflected by the fact that more than 60% of all commercially available drugs target a GPCR (more than half of all GPCRs are orphan receptors), including pharmaceuticals for cardiovascular diseases such as hypertension, arrhythmias and heart failure [20, 76]. Although cardiovascular diseases remain the leading cause of death worldwide, the majority of GPCR modulating cardiovascular drugs exert their function by targeting only the adrenergic and angiotensin GPCR signaling pathways [30, 37]. It is likely, however, that as yet uncharacterized cardiac GPCRs offer opportunities for the development of novel therapies for heart diseases. A recently performed large-scale, next-generation sequencing analysis revealed mRNA expression of a subset of “non-classical” GPCRs, e.g., chemosensory receptors such as taste receptors and odorant receptors (ORs), in the human heart [28]. The superfamily of OR genes was initially identified in the olfactory epithelium of rat as mediators of olfactory chemosensation [11]. Nevertheless, a number of human genes have been classified as OR based on sequence similarity and by the presence of certain predicted protein motifs, and they are also expressed in non-olfactory tissues [4, 25, 28, 36, 115]. Their discovery sparked a controversial discussion about the potential function of such “ectopically expressed” ORs. Many groups have reported mammalian OR expression at the transcript and/or protein level in various healthy as well as pathophysiologically altered tissues such as prostate cancer [19, 25, 31, 34, 70, 101, 105, 109, 111, 115].

Because of upregulated expression in cancer cells, the odorant receptors OR51E1 und OR51E2 were previously implicated as tumor biomarkers [19, 101]. Recently, ORs came into the focus of drug development because ectopically expressed ORs were involved in physiological and pathophysiological mechanisms within human tissues, such as the chemotaxis of sperm, the proliferation of prostate cancer and liver cancer cells, the induction of wound-healing, the modulation of hepatic triglyceride metabolism, cytokinesis, apoptosis and the regulation of serotonin secretion [8, 13, 58, 60, 67, 79, 82, 90, 99, 108, 114].

To date, the functional role of ORs in the heart is still unexplained, although OR mRNA expression in the rat heart had already been analyzed during ontogenetic development 20 years ago [23, 26]. Therefore, the aims of the present study were to analyze the cardiac OR expression profile in greater detail, leading to a special focus on the medium-chain fatty acid (MCFA)-sensing OR51E1 receptor. In this study, we characterized its physiological function in the human heart.

## Experimental procedures

### Cell culture

Human embryonic stem cells (hESCs) and human-induced pluripotent stem cells (hIPSCs) were cultured and differentiated into cardiomyocytes.

Spontaneously beating clusters were generated by co-culturing the hES cell line HES-2 [77] or the hIPS cell line Foreskin C1 [112] with inactivated visceral endoderm like cells (END-2) according to Passier et al. [71], with own modifications. Briefly, HES-2 or hiPS colonies were cultivated for 7 days on irradiated CF1 mouse feeder cells under standard conditions (Madison, WI, USA, http://www.wicell.org), detached with 0.2% Collagenase IV (w/v) and distributed into a six-well dish prepared with ~6 × 10^5^ END-2 cells. The differentiation media consisted of DMEM/F12 (+Glutamax) supplemented with 1% fetal bovine serum, 1% nonessential amino acids, 0.1 mM beta-mercaptoethanol, 50 U/ml and 50 µg/ml penicillin and streptomycin. Reagents, unless indicated otherwise, were purchased from Invitrogen (Life Technologies, Carlsbad, CA, USA). From the third week of differentiation on, the medium content of FBS was increased to 2%. Single cardiomyocytes were isolated from the beating clusters by subsequent enzymatic digestion and plated on fibronectin—(2.5 µg/ml D-PBS) and gelatin—(0.1% in D-PBS) coated coverslips for calcium measurements. The use of hESCs in this project was permitted by the Robert Koch Institute, Berlin, Germany (permission number 1710-79-1-4-2-E05). The cell line Foreskin C1 utilized in this study was provided by James A. Thomson (University of Wisconsin, Madison, WI, USA) [112]. hIPSC-derived cardiomyocytes (iCell^®^-CMs) were purchased from Cellular Dynamics International (Cellular Dynamics International, Madison, Wisconsin, USA) and maintained by the standard protocols. Single cells were plated on the coated coverslips after dissociation according to the manufacturer’s guidelines.

Hana3A cell line was kindly provided by Prof. H. Matsunami (Duke University Medical Center, Durham, NC, USA). Hana3A is an HEK293-derived cell line stably expressing RTP1L, RTP2, REEP1 and Gα_olf_, which supports the robust heterologous expression of ORs [81]. Cells were maintained in DMEM (Gibco^®^, Life Technologies) supplemented with 10% FBS and 100 units/ml penicillin and streptomycin. Stem cell-derived cardiomyocytes and Hana3A cells were maintained at 37 °C in a 5% CO_2_ humidified atmosphere.

### Human myocardial tissue culture

Myocardial tissue specimens were procured from patients undergoing heart transplantation. Patients provided informed consent to the scientific use of the explanted tissue, and the study was approved by the local ethics boards of the clinical and the experimental study contributors (Nr. 63-012). Ventricular myocardium was available from heart transplantation and collected at the Heart and Diabetes Center of NRW as 2 × 2 cm^2^ transmural biopsies of the left ventricular wall, which were immediately placed in cold (4 °C) BDM containing HEPES buffered salt solution. Samples were sent by overnight courier to the Walter Brendel Center, Munich, where 300-µm-thick tissue slices were prepared as previously described [7]. Briefly, trimmed tissue blocks measuring approximately 1 cm^3^ were embedded in 4% agarose, mounted onto a precision vibratome (Leica VT1200S; Leica, Wetzlar, Germany), and cut along the transversal direction proceeding from the endo- to epicardial layers of the myocardium. Myocardial slices were attached to permeable tissue culture membranes (Millicell-CM, Merck Millipore, Billerica, Massachusetts, USA) and were kept in culture at a tissue–air interface (5% CO_2_, 20% O_2_, 37 °C) for up to 8 days. The medium (M199 with PS and insulin–transferrin–selen supplements, serum-free; Invitrogen, Carlsbad, CA, USA) was exchanged daily. For the first day of culture, the medium was also supplemented with BDM (30 mM) to support the recovery of tissues from 18 to 28 h of transport. The viability and contractility of slices were similar to those reported for cultured hypertrophic myocardium [7].

### Transcriptome analysis

To analyze the expression profile for the most highly expressed ORs in human hearts, we used next-generation sequencing data from Flegel et al. (adult heart) and reanalyzed a raw data set from a fetal heart, which was available in the NCBI SRA archive under the GEO accession number GSM1059495 as previously described [28]. The raw sequence data were aligned to the human genome reference sequence (hg19) using TopHat [94]. FPKM (fragments per kilobase of exon per million fragments mapped) values were calculated using the Cufflinks software [95].

### RT-PCR

RNA isolation from heart tissue and stem cell-derived cardiomyocytes and the subsequent RT-PCR were preformed as previously described [60]. The temperature cycle profile was as follows: 5 min at 95 °C followed by 35 cycles of 45 s at 95 °C, 45 s at 60 °C, 45 s at 72 °C and a final extension of 10 min at 72 °C. The primers used for RT-PCR were as follows:

NANOG (5′-CAGCCCTGATTCTTCCACCAGTCCC-3′ and 5′-TGGAAGGTTCCCAGT-CGGGTTCACC-3′), TNNT2 (5′-ATGAGCGGGAGAAGGAGCGGCAGAAC-3′ and

5′-TCAATGGCCAGCACCTTCCTCCTCTC-3′), OR51E1 (5′-CTCTTCTGGAGGAAGA-CTGG-3′ and 5′-GTTACCTAGCACAGCAATAAGG-3′), GNAL (5′-CAGACCAGGACCTCCTCAGA-3′ and 5′-AGGGACTCTCTCAGCCTGTT-3′), ADCY3 (5′-AAGGATTCAACCCTGGGCTC-3′ and 5′-TCCAGCGTCGCATCTCATAG-3′), CNGA2 (5′-TACTCTGGGACCACCACTGA-3′ and 5′-AACTATCCTGCGGAAGCCAC-3′), CNGA4 (5′-GAGGTGCTGAGCGAGTATCC-3′ and 5′-CAGCCGTTCAATGCGGTAAG-3′), CNGB1 (5′-GTCTGAGGCAGCACCTGTAG-3′ and 5′-CGTAGAGAAGGTGATCCCGC-3′) and intron-spanning actin (ACTB) primer to exclude amplification from genomic DNA contamination (5′-GTCTCCCCCTCCATCGTG-3′ and 5′-TGGATGCCACAGGATTCCA-3′).

### Co-immunoprecipitation and western blot

The following primary antibodies were used: custom-made affinity purified rabbit polyclonal antibody against OR51E1 (Eurogentec; dilution: 1:250; epitope: LRLFHVATHASEP), polyclonal rabbit anti-Gα_s/olf_ antibody (Santa Cruz Biotechnology, Dallas, Texas, USA; dilution 1:250) and polyclonal rabbit anti-adenylyl cyclase III antibody (Santa Cruz Biotechnology; dilution 1:250)

Tissue samples were homogenized in lysis buffer (50 mM Tris HCl, pH 7.4, 150 mM NaCl, 1 mM EDTA, 1% Triton X-100) with a Complete^®^ protease inhibitor mixture (Roche, Basel, Switzerland) using the Precellys^®^24 (Bertin Technologies, Montigny-le-Bretonneux, France) and Precellys Ceramic Kit 1.4/1.8 (Peglab, Erlangen, Germany). Samples were centrifuged for 5 min at 14,000 rpm, and the supernatant was isolated and kept on ice. The co-immunoprecipitation experiments were carried out using the Catch & Release system (Catch & Release v2.0, Merck Millipore). For this propose the lysate was incubated with primary antibodies (each 2 µg) overnight at 4 °C in a spin column. After several washing steps, the proteins were eluted from the column using a denaturing elution buffer. Unspecific IgG was used for precipitation as control. Samples were loaded onto a SDS gel and western blot analysis was performed as described by Neuhaus et al. [67], with the slight modification that we used the ECL™ Select Western Blotting Detection System (Amersham Biosciences, GE Healthcare, Solingen, Germany) and the Fusion-SL image acquisition system (Vilber Lourmat Deutschland GmbH, Eberhardzell, Germany) for detection.

### Immunocytochemistry

The following primary antibodies were used: custom-made affinity purified rabbit polyclonal antibody against OR51E1 (dilution 1:50), monoclonal mouse anti-α-actinin (sacromeric) antibody (Sigma-Aldrich, St. Louis, Missouri, USA; dilution 1:500) and mouse monoclonal anti-rhodopsin antibody 4D2 (Abcam, Cambridge, UK; dilution 1:250).

Stem cell-derived cardiomyocytes or Hana3A cells were seeded on coverslips. Hana3A cells were transfected with rho-tagged OR51E1 plasmid as described in the section titled “Luciferase reporter assay”, and the detection of heterologously expressed OR51E1 served to control the specificity of the anti-OR51E1 antibody. The cells and human myocardial tissue slices were fixed by incubation with 4% paraformaldehyde at 4 °C for 20 min. The specimens were washed and permeabilized in PBS + Triton X-100 (PBST). Blocking was performed in PBST + 1% gelatin and 5% goat serum for 1 h at room temperature. The specimens were then incubated overnight with the primary antibody in PBST + 1% gelatin at 4 °C. After PBST washing steps, secondary fluorescent IgGs (Life Technologies) (1:1000) and 40,6-diamidino-2-phenylindole (DAPI) were used for visualization. The secondary antibody incubation occurred for 45 min at room temperature. Afterwards, cells or tissue slices were washed with PBST and covered with Prolong^®^ Gold Antifade reagent (Life Technologies). Micrographs were captured using a LSM510 Meta confocal microscope (Zeiss, Jena, Germany) with a 1024 × 1024 or a 2048 × 2048 pixel resolution.

Immunizing peptide-blocking experiments were performed to validate antibody specificity; therefore, the anti-OR51E1 antibody was preincubated with blocking peptide (Eurogentec, Seraing, Belgium) at a 1:7 ratio for 30 min at room temperature before proceeding with the staining protocol. Additional proof of the anti-OR51E1 antibody specificity is provided by Maßberg et al. [59]. However, it should be noted that unspecific binding of the antibody, when tested in heart tissue, cannot be completely excluded due to missing knock-down or knock-out controls.

### siRNA transfection

Stem cell-derived cardiomyocytes were transiently transfected with either targeted or negative control siRNAs (OR51E1 Silencer^®^ Pre-designed siRNA s44550 and Silencer^®^ Select Negative Control No. 1 siRNA; Life Technologies) using Lipofectamine^®^ RNAiMAX (Life Technologies) according to the manufacturers’ instructions. The transfection rates were less than 1% with both OR51E1 siRNA and control siRNA as detected by co-expression of GFP.

### Luciferase reporter assay

The Dual-Glo Luciferase Assay System (Promega, Madison, Wisconsin, USA) is the most commonly used method for high-throughput screening of odorant receptor pairs [14, 56, 75, 86, 113]. This method quantifies cellular responses as an indirect measure of odorant receptor activation as previously described [117]. Hana3A cells seeded on a 96-well plate (Thermo Fisher Scientific, Waltham, Massachusetts, USA) were transfected at 60–70% confluence with Lipofectamine 2000 (Life Technologies) using 18 µl Lipofectamine, 1 µg of RTP1S plasmid [116], 1 µg of pRL-TK-*Renilla* (Promega), 2 µg of pGL4.29-luciferase (Promega), 1 µg of hM3 [51] and 5 µg of full-length rho4D2-tagged OR51E1 in pCI (Addgene Cambridge, Massachusetts, USA) for an entire well plate. Approximately 18–24 h after transfection, the transfection medium was removed and replaced with the appropriate concentration of odorant, diluted in DMSO, 0.1% DMSO (negative control) or 10 µM forskolin (positive control) in CD293 (Life Technologies) with 2 mM l-glutamine. Most of the odorants were provided as a generous gift from Dr. J. Panten (Symrise, Holzminden, Germany; Table S1). Four hours after odor stimulation, luminescence was measured using a Fusion microplate reader (Packard BioScience PackardBioScience, Meriden, Connecticut, USA). Firefly luminescence values were divided by the *Renilla* luciferase activity as a control for transfection efficiency in a given well. The firefly–*Renilla* luciferase ratio was normalized against the lowest/highest luciferase ratios obtained for that experiment. Normalized luciferase activity was calculated by the formula [Luc/Ren(N) − Luc/Ren(lowest)]/[Luc/Ren(highest) − Luc/Ren(lowest)], where Luc/Ren(N) is the luminescence of firefly luciferase divided by the luminescence of *Renilla* luciferase in a certain well; Luc/Renilla(lowest) is the lowest luciferase ratio of OR51E1 transfected cells to negative control; Luc/Ren(highest) is the maximum luciferase ratio of OR51E1 transfected cells to forskolin or nonanoic acid (1000 µM) of a plate. Mock-transfected cells were stimulated to exclude unspecific responses to the tested compounds. Data were analyzed using Microsoft Excel® (Microsoft, WA, USA) and SigmaPlot (Systat Software Inc., San Jose, CA, USA).

### Ca^2+^ imaging

Stem cell-derived cardiomyocytes plated on glass were incubated for 25 min in loading buffer (pH 7.4) containing Ringer’s solution (140 mM NaCl, 5.9 mM KCl, 10 mM HEPES, 2 mM CaCl_2_, 1 mM MgCl_2_, 10 mM glucose and 2 mM Na-pyruvate) and 7.5 μM Fura-2-AM (Life Technologies, Carlsbad, CA, USA). After removal of extracellular Fura-2 by washing with Ringer’s solution, ratiofluorometric Ca^2+^ imaging was performed using a Zeiss inverted microscope equipped for ratiometric imaging and a Polychrome V monochromator (TILL Photonics, Graefelfing, Germany). Images were acquired at 10 Hz, and integrated fluorescence ratios (*f*_340_/*f*_380_) were measured using TILLvisION software (TILL Photonics). Cells were visualized with a 20 × objective (UPLSAPO, Olympus, Tokio, Japan). Images were acquired in randomly selected fields of view. Carbachol and inhibitors were purchased from Sigma-Aldrich or Tocris (R&D Systems, Minneapolis, MN, USA). Odorants were prediluted in DMSO (Sigma-Aldrich) and then diluted in Ringer’s solution such that the DMSO concentration did not exceed 0.1% (v/v), which was well tolerated by cardiomyocytes. The data for Fura-2 calcium transients were analyzed with a self-written script in Spike2^®^ analysis software (Cambridge Electronic Design, Cambridge, UK) and partly by Chart5^®^ (ADInstruments, Oxford, UK, http://www.adinstruments.com). Basic statistical analysis was performed in Excel^®^ and Sigmaplot.

### Contractile force measurements of slice preparations of adult human ventricle

Myocardial slices with a surface area of approximately 5 × 5 mm^2^ were mounted onto a horizontal organ bath (Mayflower, Hugo Sachs Elektronik, March, Germany) and were superfused with gassed Ringer solution (5% CO_2_, 20% O_2_, 37 °C) at 4 ml/min as previously described [7]. The isometric contraction force was measured at a preload of 1.5 mN under continuous field stimulation (rate 0.5/s, pulse duration 3 ms) at a 1.5-fold excitation threshold. The relative alteration of the twitch force before and after drug application was evaluated. For drug application, perfusion of the organ bath was stopped and fatty acids dissolved in DMSO were added at 0.1% v/v to the organ bath. Increasing concentrations of the same fatty acid were tested in sequential applications, which were separated by 4-min intervals of perfusion and equilibration. Preparations that developed a twitch force of less than 0.4 mN or reacted by more than a 5% change in contractility to 0.1% DMSO were discarded. Substances that caused a change of less than 5% in the twitch force at their maximum concentration (1 mM) were considered inactive. Because of the transient action of some of the fatty acids, the acids’ effects on the twitch force were analyzed as minimum and final values over a 4-min period of exposure.

### Contractile force measurements of trabeculae carneae

Within 2 h after explantation, trabeculae carneae of the left ventricle were prepared and fixed in a horizontal organ bath setup. Contractions of the muscle were induced by an STI-08 stimulator at a rate of 1 Hz. Diastole and systole were detected via mechanical transducers and an FMI TMI-1020-Shor amplifier. Signals were recorded with BEMON FMI VitroDat 3.4 software (Föhr Medical Instruments GmbH, Seeheim, Germany). Trabeculae carneae were submerged in warm (37 °C) and oxygenated physiological buffer solution with a precisely controlled pH of 7.4. Prior to the beginning of each experiment, the contraction force of the trabeculae carneae was maximized by raising the tension to achieve an optimal preload (Frank–Starling mechanism). Because overstretching leads to damage of the contractile elements and a subsequent loss of contraction force, the tension was raised in small, carefully controlled steps. After optimizing the applied tension, the application of substances was delayed until a steady contraction force was observed. Substances were directly added to the bath solution. At the beginning of the measurements, 5 × 10^−8^ M isoprenaline (Sigma-Aldrich), a beta-receptor agonist, was applied to the organ bath to further elevate the contraction force of the muscle. Following this prestimulation, increasing concentrations of the OR51E1 ligand, nonanoic acid, were applied to the bath solution. The administered concentration started at 1 μM, with the concentration increased fivefold every 5–10 min until 5 mM was reached. Meanwhile, changes in the contraction force were closely monitored. To exclude possible toxic effects of nonanoic acid, stimulation with 1 mM isoprenaline was performed after each experiment.

### Determination of the fatty acid pattern in the triglyceride and non-esterified fatty acid fraction from epicardial adipose biopsies and from plasma

Approximately 25 mg of human epicardial adipose tissue was homogenized using a TissueLyser MM 300 (Qiagen, Hilden, Germany) in 0.25 ml 1% Triton X-100 in PBS. Then, 1.25 ml 2-propanol, *n*-heptane and 2 mol/l phosphoric acid (40:20:1 by vol) were added to the tissue extract or to 0.25 ml plasma samples and mixed by vortexing. After 10 min, 0.5 ml toluene/methanol (4:1 by vol) and 0.75 ml water were added and mixed by vortexing, and after centrifugation at 4000 rpm (8175 *g*), the upper phase was dried under stream of nitrogen. The lipids were dissolved in 75 µl CHCl_3_/CH_3_OH (2:1 by vol) and applied to a silica gel chromatography plate (Merck, Darmstadt, Germany). The lipid fractions were separated using a mixture of *n*-hexane, diethylether and acetic acid (160:40:6 by vol) as a solvent. The lipid fractions were identified using a pooled control plasma and were separated on each plate; the lipid fractions were then visualized by 2,7-dichlor-fluoresceine under ultraviolet light. The fractions were scraped off the TLC plate, transferred to screw-capped vials and dissolved in a 2 ml methanol/toluene mixture (1:4 by vol) containing *cis*-13,16,19-docosatrienoic acid (10 µg/ml) as an internal standard. Trans-esterification was performed by incubation with acetyl chloride at 100 °C for 60 min. The cold sample was neutralized with 5 ml 6% K_2_CO_3_, shaken for 2 min, and centrifuged, and the upper phase was concentrated to 100 μl under nitrogen. The fatty acid methyl esters were measured by gas chromatography 7890A with a flame ionization detector (Agilent, Waldbronn, Germany) and quantified using the corresponding fatty acids standards. All data analysis were performed using the JMP 11.0 software package (SAS Institute, Cary, NC, USA).

## Results

### Olfactory receptor OR51E1 is expressed in the human heart and in stem cell-derived cardiomyocytes

**Fig. 1:**
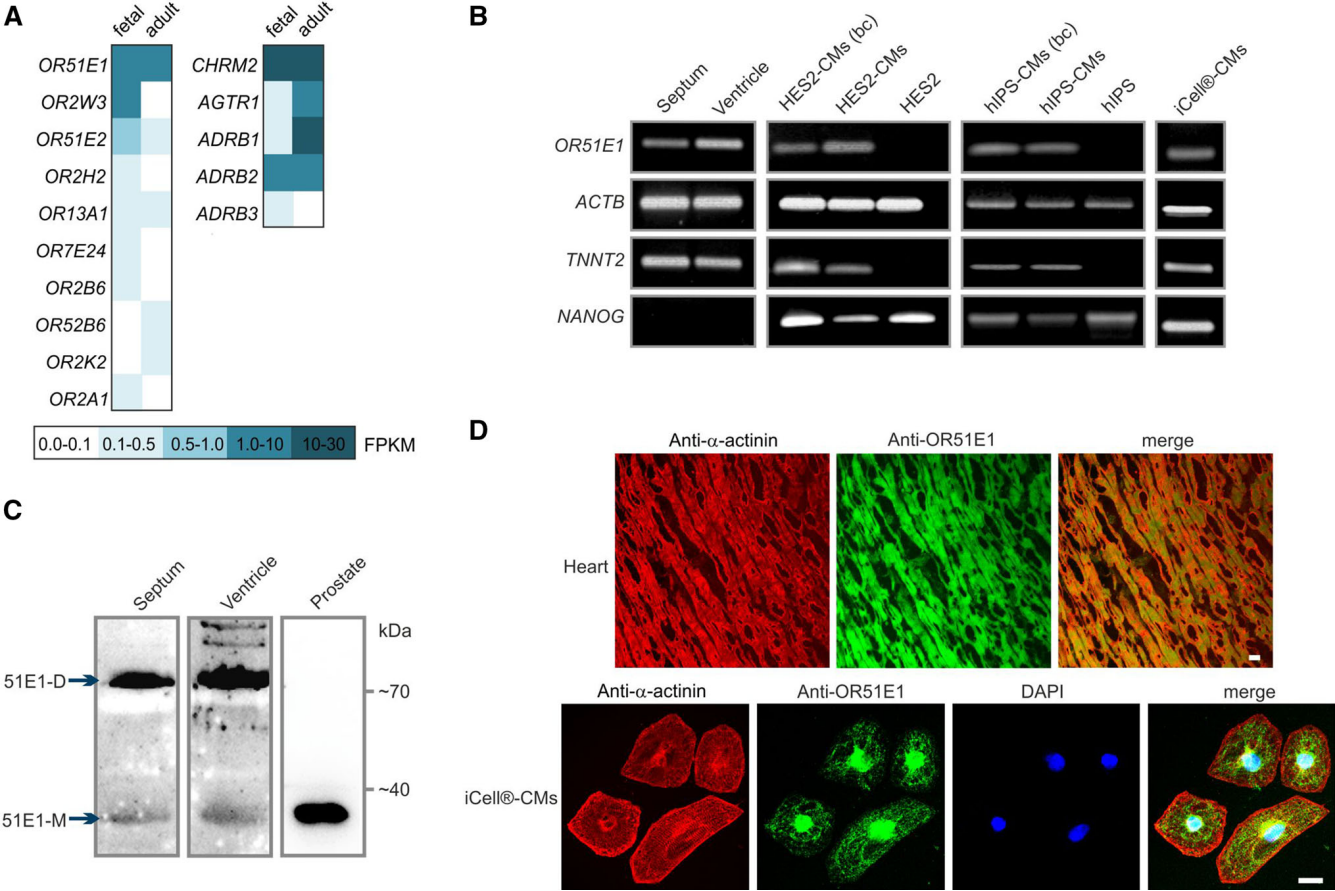
Expression of OR51E1 in the human heart tissue and stem cell-derived cardiomyocytes. **a** Expression pattern of ORs as revealed by next-generation sequencing analysis. The heat map shows FPKM values for ORs and classical cardiac GPCRs (CHRM2, muscarinic acetylcholine receptor M_2_; AGTR1, angiotensin II receptor type 1; ADRB1, beta-1 adrenoceptor; ADRB2, beta-2 adrenoreceptor; ADRB3, beta-3 adrenoreceptor) found in human adult and fetal heart tissue analyzed. *Dark aquamarine* indicates high expression (FPKM values higher than 10), and *white indicates* the absence of detectable transcripts. **b** Detection of *OR51E1* transcripts in the left ventricle and septum of explanted human heart and in stem cell-derived cardiomyocytes by RT-PCR. Amplification of β-actin (*ACTB*) using intron-spanning primers served to control cDNA quality. Cardiac muscle troponin T (*TNNT2*) expression identifies induced cardiomyocytes (CMs) and nanog homeobox (*NANOG*) expression undifferentiated stem cells. HES2-CMs: human embryonic stem cell-derived cadiomyocytes, hIPS-CMs: human induced pluripotent stem cell-derived cardiomyocytes, (bc) indicates beating cell clusters in stem cell-derived cardiomyocytes that were excised for RNA preparation under optical control. *OR51E1* is expressed in cardial myocytes of embryonic and adult stem cell origin, not in undifferentiated stem cells. **c** Detection of OR51E1 protein in the left ventricle and septum of explanted human heart and prostate as control by western blotting, the size of the OR51E1 monomeric (51E1-M) protein is 35 kDa and dimeric (51E1-D) 70 kDa. **d** Detection of OR51E1 protein in ventricular myocytes and stem cell-derived cardiomyocytes by immunohistochemical staining. Shown are confocal micrographs of OR51E1 immunostaining obtained using an OR51E1-specific antibody in transversal cryosections of human left ventricle. Cardiomyocytes were identified by co-staining with an α-actinin-detecting antibody. The *lower panel* shows immunostaining of stem cell-derived cardiomyocytes with anti-α-actinin and anti-OR51E1 antibodies. Cell nuclei were stained with 4′,6-diamidino-2-phenylindole (DAPI). *Bar* indicates 20 µm

Comparative transcriptome analysis of OR expression identified *OR51E1* in various human tissues, including the heart [28, 32]. In the human adult and fetal heart, *OR51E1* is the highest expressed OR (adult 1.50 FPKM; fetal 1.35 FPKM), with an expression level similar to that of the beta-2 adrenergic receptor and the muscarinic acetylcholine receptor M2 (Fig. 1a). On a rough scale, 1 FPKM corresponds to a weak expression level, 10 FPKM represents a moderate expression level and   100 FPKM indicates a high expression level. Moreover, OR51E1 is one of the few ORs for which the ligand (nonanoic acid) has been identified [1, 80]. Therefore, in this study, we focused on OR51E1 for functional characterization of ORs in the human heart. First, we validated the results of the transcriptome analysis using reverse transcription PCR (RT-PCR) and could detect transcripts of *OR51E1* in the investigated septum and ventricle of the human heart (Fig. 1b). For the detection of OR51E1 receptor proteins, we performed western blotting and immunohistochemical analysis using a custom-made OR51E1 antibody. The antibody specificity was demonstrated by co-immunocytochemical staining of Hana3A cells heterologously expressing rho-tagged OR51E1 (see supplementary Figure S1A) and by using of a specific OR51E1-blocking peptide (Figure S2). Western blot analysis revealed OR51E1 protein expression in the human septum and ventricle. Prostate cancer tissue served as a positive control for the detection of OR51E1 protein [101, 105] (Fig. 1c). Immunohistochemical analysis of human ventricular tissue sections further confirmed our results regarding myocardial OR51E1 protein expression. To study receptor activation, we used an established cardiac in vitro model, hIPSCs and hESC-derived cardiomyocytes that are spontaneously electrically active [92]. The expression of OR51E1 mRNA in different stem cell-derived cardiomyocytes was confirmed by RT-PCR and the subcellular localization of OR51E1 was elucidated by immunocytochemical staining (Fig. 1b, d).

### Ligand screening on OR51E1

**Fig. 2:**
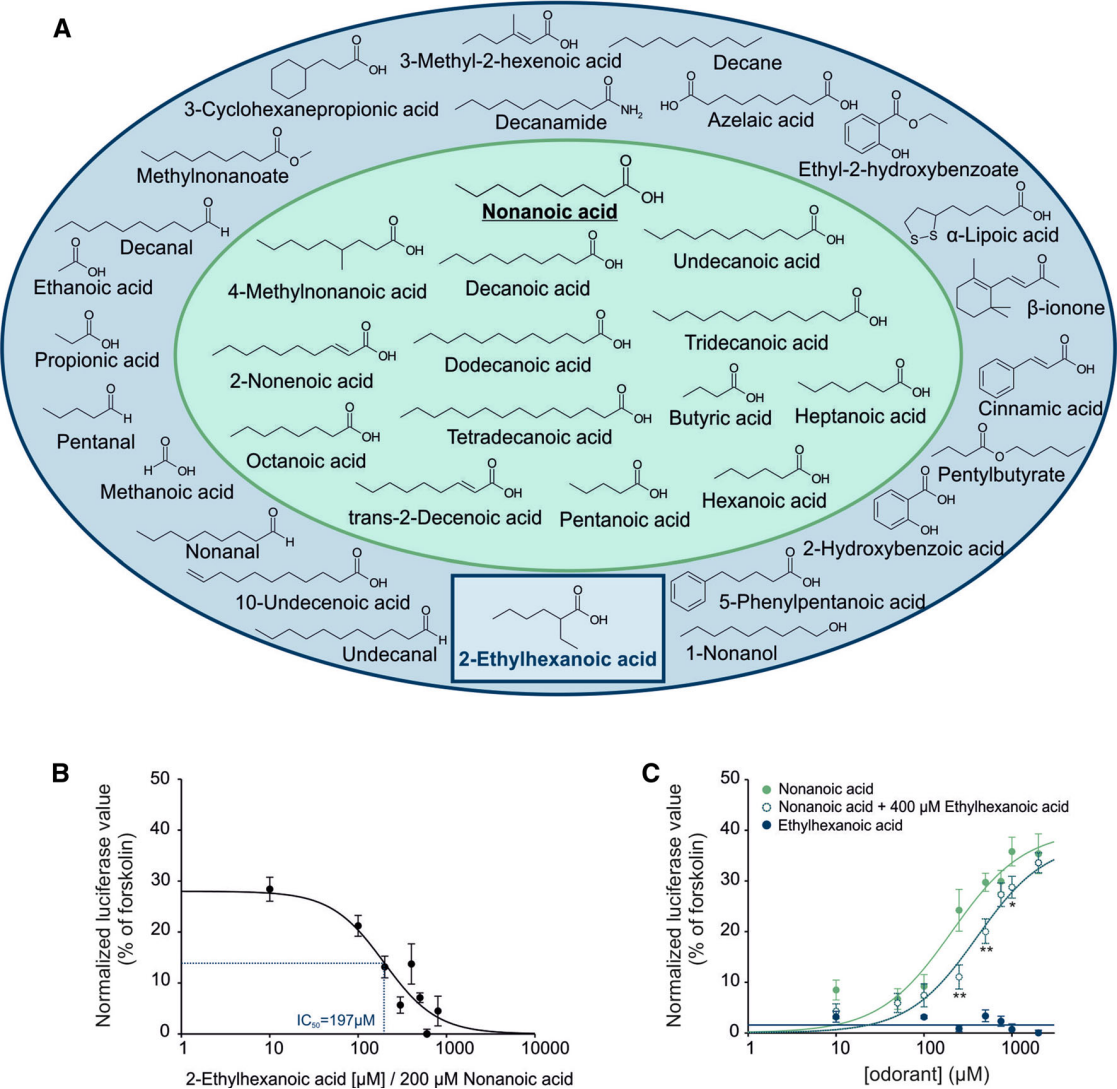
Ligand spectrum of OR51E1. **a** Molecular receptive field of OR51E1. Structurally related molecules were tested in luciferase reporter assays for their ability to activate heterologously expressed OR51E1, using nonanoic acid as a template. Effective ligands are shown in a *green field*; inactive compounds are in a *blue field*. Receptor-inhibiting substance is shown in a *blue rectangle*. The active analog approach identifies a mono carboxyl functional group as key feature for OR51E1 activation; alkyl side chain length can vary from C4 to C14; methyl substitutions are not tolerated at Cβ. Double bonds are tolerated at Cβ but affect negatively the activating property of the compound at Cκ (e.g., undecanoic acid: activating, 10-undecenoic acid: inactive). Aromatic functional groups are not tolerated. **b** Concentration–inhibition curve. Each response was normalized to the agonist nonanoic acid (200 µM) alone. The calculated IC_50_ was 192 μM. The curve shift was significant at concentrations higher than 100 µM 2-ethylhexanoic acid (*p < 0.05). **c** Dose–response curves in the presence of OR51E1 antagonist. To determine receptor-inhibiting properties of compounds, OR51E1-expressing cells were co-stimulated with a rising concentration of nonanoic acid and a fixed concentration of 2-ethylhexanoic acid (400 µM). The mean of cellular responses was measured by luciferase reporter assays in four biological replicates and normalized to the positive control (forskolin). 2-ethylhexanoic acid acts as a competitive inhibitor on OR51E1. Significance was calculated by Student’s *t* test (*p < 0.05, **p < 0.01 and ***p < 0.001). *Error bars* represent the SEM

Previous de-orphanization studies have identified *inter alia* nonanoic acid as an activating ligand for OR51E1 [1, 80] (Figure S3A, B, D). To characterize the molecular receptive field of OR51E1 in greater detail, we conducted luciferase reporter assays using Hana3A cells heterologously expressing OR51E1 and based on the known ligand nonanoic acid we test further 36 structurally related odorant on their ability to activate the receptor (Table S1). We identified 13 new agonists by testing at an odorant concentration of 500 µM (except poorly soluble substance) to ensure receptor activation above the detection threshold (Fig. 2a; Figure S3). Moreover, we identified an antagonist for OR51E1. OR51E1-activating substances are short- to middle-chain (C4–C14) saturated or monounsaturated acids (Fig. 2a), some of which are dietary fats. Among the tested compounds, decanoic acid (C10:0) appears to be the most efficient agonist (Figure S3C; EC_50_ 190 µM), whereas an increasing or decreasing chain length and insertion of branches or additional double bonds into the compound resulted in a reduced potency to activate the receptor (Fig. 2a; Figure S3). Furthermore, our results indicate that the presence of one free terminal carboxyl group is crucial for receptor recognition because substitution by aldehyde, ester, amide or alcohol groups abolished activation of the heterologously expressed receptor. In a screen for inhibitors, we identified 2-ethylhexanoic acid as an antagonist of OR51E1, which significantly reduced the nonanoic acid-induced luminescent signal. The half maximal inhibitory concentration (IC_50_) of 2-ethylhexanoic acid was calculated with 179 μM (using a concentration of 200 µM nonanoic acid) (Fig. 2b). We observed a significant shift in the dose–response curve for nonanoic acid with and without the antagonist. The EC_50_ value shifted significantly from 215 µM (± 16) to 375 µM (± 41) (*p* = 0.011). The OR51E1 activity induced by the saturating nonanoic acid concentration of 2 mM was not significantly reduced in the presence of 2-ethylhexanoic acid (400 µM), which indicates, together with the parallel shift in the dose–response curve, a competitive antagonistic mechanism (Fig. 2c).

### OR51E1 activation induces a negative chronotropic effect in human stem cell-derived cardiomyocytes

**Fig. 3:**
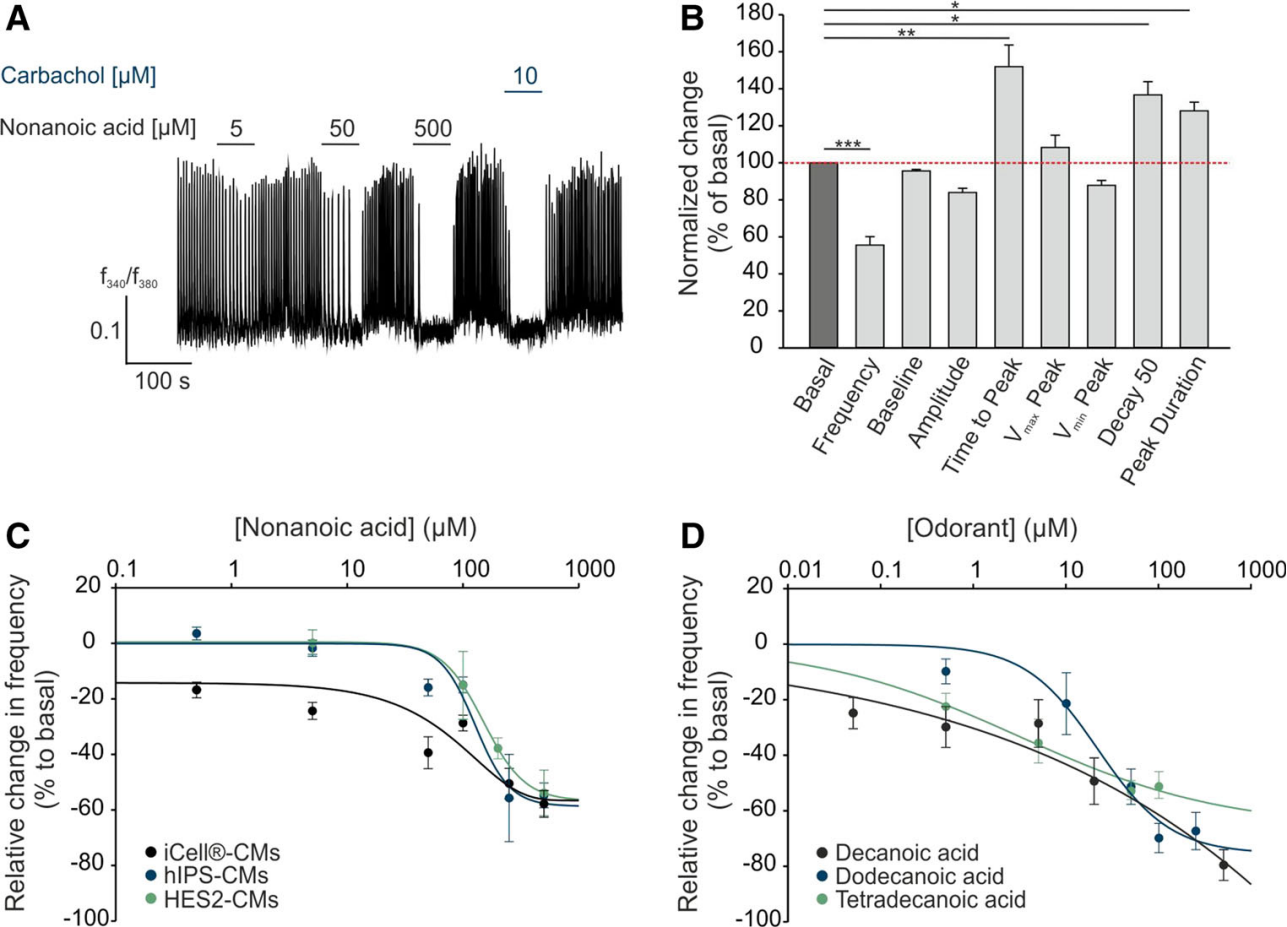
OR51E1-activation induces negative chronotropic effects in stem cell-derived cardiomyocytes. **a** Representative Ca^2+^ imaging trace of a Fura-2-loaded human-induced pluripotent stem cell-derived cardiomyocyte (hIPS-CM). Cytosolic Ca^2+^ levels were monitored as the integrated *f*_340_/*f*_380_ fluorescence ratio expressed as a function of time. *Horizontal bars* indicate time and duration of stimulus application. In a randomly selected field of view, application of nonanoic acid inhibited spontaneous Ca^2+^ transients in a dose-dependent manner. Carbachol (10 µM) served as positive control for negative chronotropy. **b** Statistical analysis of the effect of nonanoic acid (500 µM) on intracellular Ca^2+^ dynamics of cardiomyocytes. Relevant parameters of the Ca^2+^ transients during stimulus application were quantified by Spike2 and normalized to the basal value. Analysis of the resulting Ca^2+^ transients revealed that the frequency, time to peak, decay 50 and peak duration were significantly altered during nonanoic acid stimulation. The mean baseline, amplitude, *v*_max_ peak and *v*_min_ peak remain unchanged. Means were averaged from 25 to 37 experiments. *Error bars* represent the SEM. Significance was calculated by Student’s *t* test or Mann–Whitney *U* test (*p < 0.05, **p < 0.01 and ***p < 0.001). **c** Nonanoic acid-induced negative chronotropy is cell type-independent. Ca^2+^ spike frequency of iCell^®^-CMs, hIPS-CMs and HES2-CMs decreases in a dose-dependent manner up to approximately 60%. The *graph* shows the percentage change in the frequency from the basal value. Means were averaged from 5 to 37 experiments. **d** Negative chronotropic effect of iCell^®^-CMs to OR51E1 ligands: decanoic, dodecanoic and tetradecanoic acid. The data are shown as the mean ± SEM (*n* > 29)

We next investigated the physiological function of OR51E1 activating ligand nonanoic acid [1, 80] on calcium handling in stem cell-derived cardiomyocytes via the Ca^2+^ imaging method. Interestingly, short-term application (1 min) of nonanoic acid inhibited spontaneous Ca^2+^ transients in a dose-dependent manner. The muscarinic acetylcholine receptor agonist carbachol served as a positive control for a negative chronotropic effect [39, 106] (Fig. 3a). Detailed statistical analysis of the intracellular Ca^2+^ dynamics of stem cell-derived cardiomyocytes revealed that nonanoic acid significantly reduced the frequency of Ca^2+^ spikes and increased the time to peak, decay 50 and peak duration, whereas other parameters (baseline, amplitude, *v*_max_ peak and *v*_min_ peak) remained unaffected (Fig. 3b). Dose–response curves showed that nonanoic acid reduced the frequency of Ca^2+^ spikes down to 60% compared with the basal frequency in all three tested stem cell-derived cardiomyocyte types (EC_50_ 151 ± 12 µM) (Fig. 3c). We next analyzed the effect of other OR51E1 agonists on iCell^®^ cardiomyocytes. We tested decanoic, dodecanoic and tetradecanoic acid in Ca^2+^ imaging experiments and observed that all three fatty acids induced a negative chronotropic effect in human stem cell-derived cardiomyocytes in a dose-dependent manner (Fig. 3d). Dodecanoic and tetradecanoic acid could only be used at low concentrations because higher concentrations were incompletely soluble. Notably, diluted OR51E1 ligands did not affect the neutral pH of the applied solutions at the tested concentrations, and the solvent (DMSO) did not exhibit any effect when applied alone (Figure S4A). Compounds that were inactive on the heterologously expressed OR51E1, such as propionic or cinnamic acid, did not affect the Ca^2+^ spike frequency of stem cell-derived cardiomyocytes (Figure S4B). Thus, the receptive field of heterologously expressed OR51E1 was in accord with the ligand profile observed in stem cell-derived cardiomyocytes.

**Fig. 4:**
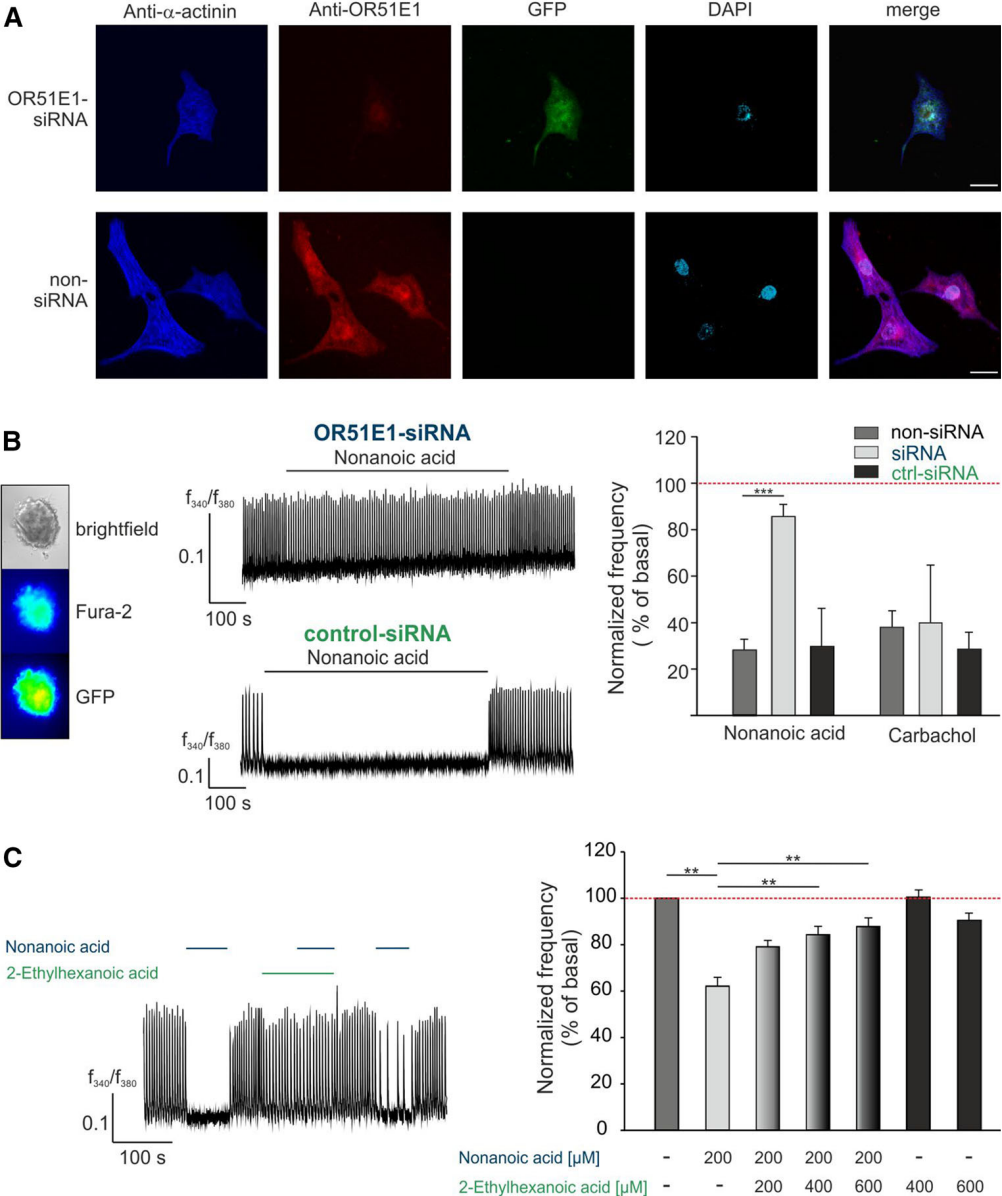
OR51E1 dependent induction of a negative chronotropic effects in stem cell-derived CMs by nonanoic acid. **a** Knock-down of OR51E1 was verified by immunostaining. HES2-CMs were transfected with siRNA directed against *OR51E1*. Because siRNA was co-transfected with a plasmid encoding for GFP, siRNA-expressing cells could be identified via GFP fluorescence. Immunostaining of HES2-CMs with anti-α-actinin (*blue*), anti-OR51E1 (*red*) antibodies and DAPI (4′,6-diamidino-2-phenylindole) staining (*turquoise*) was used to determine the number and location of cells. Control staining was performed without siRNA transfection (*lower panel*). *Bar* indicates 20 µm. **b** Ca^2+^ imaging experiments with siRNA-transfected HES2-CMs. Because the siRNA- or scRNA-constructs were co-transfected with GFP-plasmid, siRNA/sc-RNA-expressing cells could be detected via GFP fluorescence (*left panel*). The *middle panel* shows representative Ca^2+^ traces of siRNA-transfected cardiomyocytes. Nonanoic acid-induced negative chronotropy was abolished in OR51E1-siRNA expressing hIPS-CMs (*right panel*, *blue*) compared with scrambled OR51E1-siRNA (ctrl-siRNA) expressing cells (*right panel*, *green*). Carbachol (10 µM) served as a positive stimulus to control cell viability. *Bars* represent the means of 12 independent transfection experiments, and *error bars* represent the SEM. Significance was calculated by Student’s *t* test (***p < 0.001). Negative chronotropy induced by OR51E1 agonist resulted from OR51E1 activation. **c** Co-application of the OR51E1 antagonist 2-ethylhexanoic acid prevents nonanoic acid-induced negative chronotropic effect in a Ca^2+^ imaging measurement of HES2-CMs. The blocking effect was reversible because nonanoic acid-induced negative chronotropy was restored after washout of the antagonist (*left panel*). Quantification of Ca^2+^ spike frequency of co-stimulated CMs with a constant concentration of nonanoic acid and a rising concentration of the antagonist in Ca^2+^ imaging experiments (*right panel*). The data are shown as the mean ± SEM (*n* = 14–30). Significance was calculated by Student’s *t* test (**p < 0.01)

We confirmed by antagonist and receptor knock-down experiments that the observed effect of nonanoic acid primarily depends on OR51E1. For this purpose, we used RNAi to silence endogenously expressed OR51E1 in stem cell-derived cardiomyocytes. First, we investigated the knock-down efficiency via immunocytochemical staining. Cardiomyocytes were transfected with small interfering siRNA or scrambled scRNA as a negative control and a GFP-plasmid as a transfection control. Immunocytochemical analysis verified a reduction in the receptor expression level in vitro (Fig. 4a). Subsequently, we performed Ca^2+^ imaging experiments on RNAi expressing cardiomyocytes. The nonanoic acid-induced reduction of the Ca^2+^ spike frequency effect was quantified, whereby the results of siRNA- and scRNA-expressing cells were compared with those of non-transfected control cells within the same experiment. The OR51E1-specific siRNA knockdown resulted in a significant reduction of the nonanoic acid-induced negative chronotropic effect, whereas the carbachol response remained unaffected (Fig. 4b). To provide further evidence for the receptor dependence of the observed nonanoic acid-induced effect, we performed OR51E1 antagonist experiments. Co-application of antagonist 2-ethylhexanoic acid reversed nonanoic acid-induced negative chronotropy, whereas the application of the antagonist alone did not affect the Ca^2+^ spike frequency of stem cell-derived cardiomyocytes (Fig. 4c). We, therefore, concluded that the observed negative chronotropic effect results from OR51E1 activation.

### OR51E1 signaling involves G protein activation

**Fig. 5:**
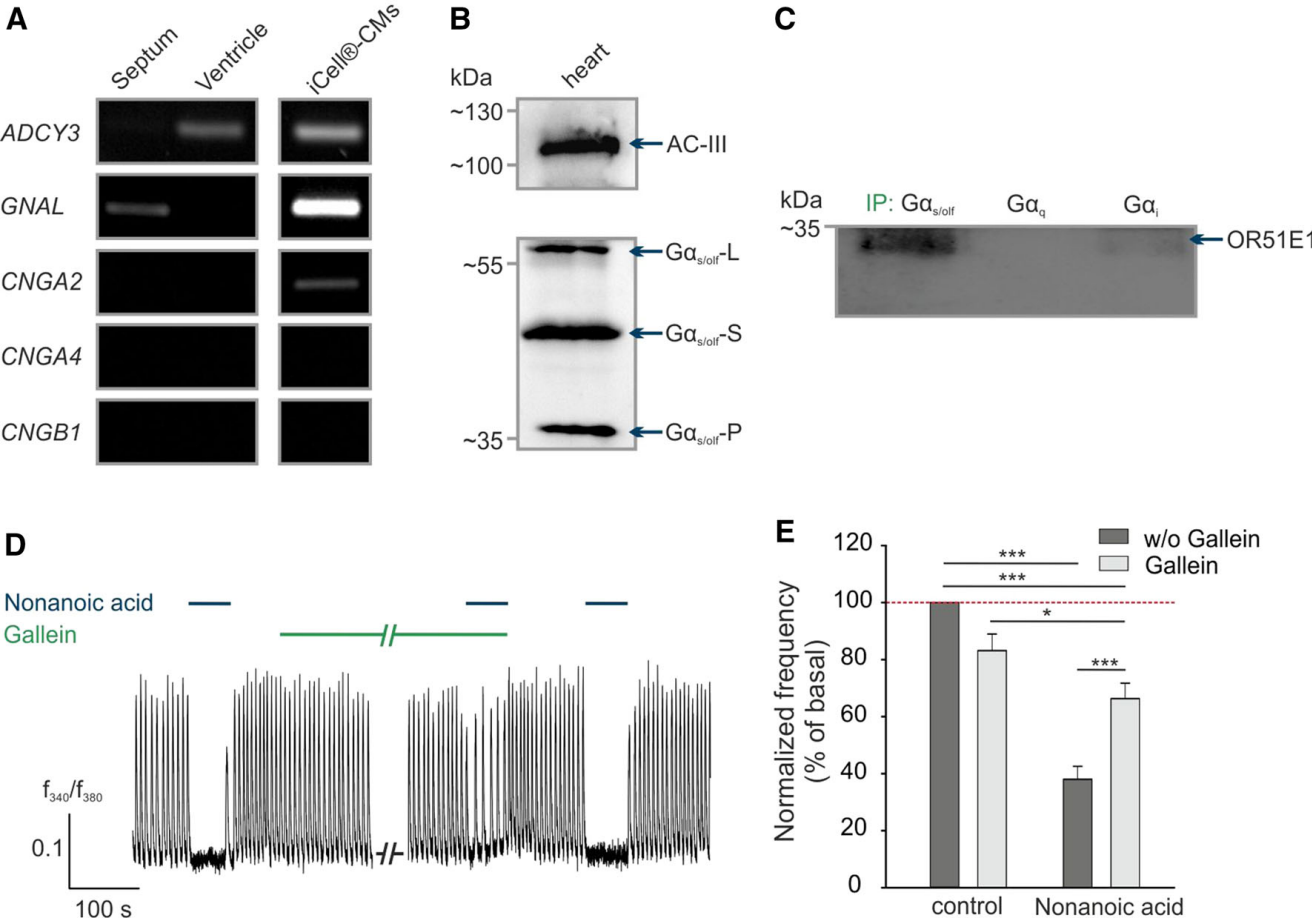
OR51E1 signaling in CMs. **a** Detection of transcripts of OR-signaling pathway components, including Gα_olf_ (*GNAL*), adenylyl cyclase III (*ADCY3*) and CNG channel subunits (*CNGA2*, *CNGA4* and *CNGB1*) in ventricle and septum of explanted human heart and iCell^®^-CMs by RT-PCR. **b** Verification of Gα_olf_ protein and AC-III expression in human heart tissue by western blot. The molecular size of the two splice variants of Gα_s/olf_ are 45 kDa (short form; Gα_s/olf_-S) and 52 kDa (long form; Gα_s/olf_-L). The 39 kDa band (Gα_s/olf_-P) may represent the proteolytic fragment of Gα_s/olf_ [35]. **c** Co-immunoprecipitations of different G protein alpha-subunits (Gα_s/olf_, Gα_q_, Gα_i_) with OR51E1 protein. The human heart lysate was immunoprecipitated (IP) with one of the indicated G proteins and detected with antibodies against the OR51E1 protein. **d** Representative recordings of Fura-2 loaded iCell^®^-CMs. Co-application of nonanoic acid (500 µM) and inhibitor gallein (10 µM). **e** Quantification of nonanoic acid**-**induced negative chronotropic effects by pretreating with or without gallein (10 µM) in Ca^2+^ imaging experiments. The data are shown as the mean ± SEM (*n* > 40). Significance was calculated Student’s *t* test or Mann–Whitney *U* test (*p < 0.05, **p < 0.01 and ***p < 0.001)

We next investigated the OR51E1-induced signaling mechanism in cardiomyocytes. In olfactory sensory neurons, the OR-activated signal transduction cascade involves the Gα_olf_ subunit, adenylyl cyclase III (AC-III) and the cyclic-nucleotide gated (CNG) channel (subunits CNGA2, CNGA4 and CNGB1) [62], whereas in ectopic tissues OR signaling can occur independently of G proteins [89]. RT-PCR results revealed that the Gα_olf_ (*GNAL*) is expressed only in the human septum and AC-III (*ADCY3*) in the human ventricle, whereas olfactory CNG channel subunits were not detected at all (Fig. 5a). Protein expression of both Gα_s/olf_ and AC-III was confirmed by western blotting in human heart tissue (Fig. 5b). In stem cell-derived cardiomyocytes, which represent a population of ventricular, atrial, and nodal cells, we observed the RNA expression of three members of the canonical olfactory pathway, *GNAL*, *ADCY3* and *CNGA2*, whereas olfactory *CNGA4* and *CNGB1* mRNA expression was not observed. By comparing expression profiles, we concluded that the OR51E1-initiated signal transduction mechanism in cardiomyocytes differs from the canonical olfactory signaling pathway. However, we aimed to elucidate which G protein couples to OR51E1 in the human heart and could show that Gα_s/olf_ interacts with OR51E1 by performing co-immunoprecipitations. The antibodies against different Gα subunits pulled down protein complexes from lysates of the isolated human heart tissue. The western blots revealed that OR51E1 protein was co-precipitated with Gα_s/olf_ protein. A weak band for OR51E1 is also visible in the Gα_i_ sample (Fig. 5c).

We next aimed to pharmacologically characterize in vitro the nonanoic acid-induced signaling that triggers the observed negative chronotropic effect.

In pacemaker cells of the human heart, stimulation of the muscarinic acetylcholine receptor M_2_ by acetylcholine or carbachol mediates negative chronotropic and inotropic effects by coupling to a PTX-sensitive G protein (G_i_/G_o_), which results in the inhibition of adenylyl cyclase and thereby a decrease in cAMP level and protein kinase A phosphorylation [78]. Furthermore, the G_βγ_ subunit of the G protein activates G protein regulated inward-rectifier potassium channels (GIRK), which cause a hyperpolarization of the cell membrane and move the sinoatrial node further from depolarization [103]. Thus, it takes longer for HCN channels to depolarize the cell, resulting in a reduced heart rate. Using the stem cell-derived cardiomyocyte model, we excluded the notion that nonanoic acid activates the muscarinic receptor pathway because the G_i_/G_o_ protein inhibitor pertussis toxin showed no effect on the nonanoic acid-induced negative chronotropic effect in Ca^2+^ imaging experiments (Figure S4C). However, gallein, an inhibitor of G protein βγ subunit-dependent signaling, significantly abolished the nonanoic acid-induced effect, likely also via GIRK (Fig. 5d, e). This finding suggests the involvement of the G protein βγ subunit and thus confirms a G protein-depended pathway induced by OR51E1 in cardiomyocytes. Further Ca^2+^ imaging measurements under calcium-free conditions or with different established inhibitors of typical GPCR signaling effectors, such as the adenylyl cyclase (MDL-12330A) or phospholipase C (U-73122), affect spontaneous Ca^2+^ transients, and we were therefore not able to draw any conclusion regarding the involvement of either AC or PLC in the OR51E1-initiated pathway (Figure S4D) [44].

### OR51E1 agonists reduce contraction force of explanted heart preparations

**Fig. 6:**
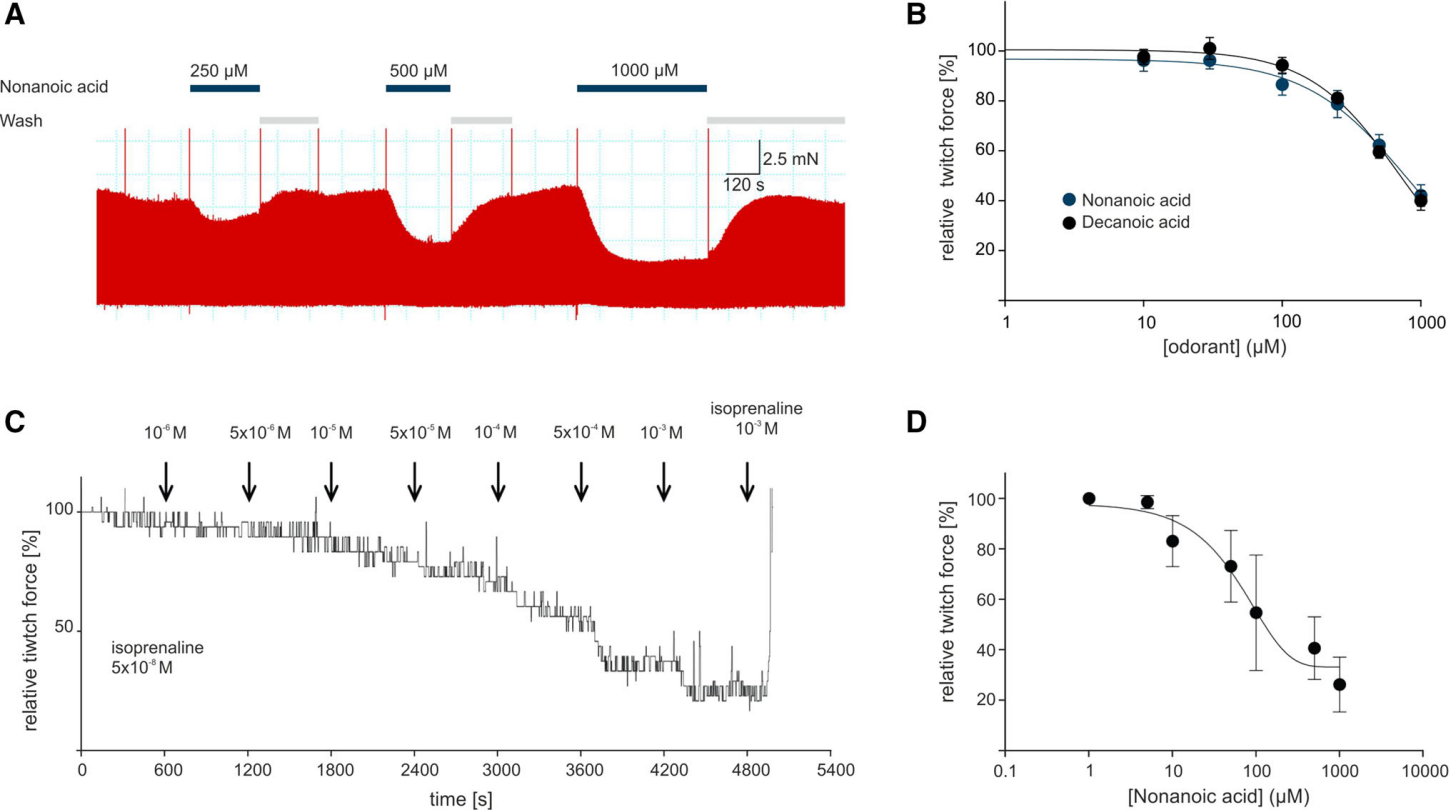
Nonanoic acid induces transient negative inotropic effects in preparations of explanted human ventricles. **a** Representative recording of a contractile force measurement on slice preparations with OR51E1 agonist nonanoic acid. Nonanoic acid stimulation repetitively induced a dose-dependent decrease in contractile force of slice preparations (300 µm thickness) of adult human failing ventricle, which was reversible after washout. The twitch force was measured at 0.5 Hz electrical stimulation (3 ms, 50% above threshold). Application of nonanoic acid is indicated by *blue horizontal bars*. **b** The dose dependence of nonanoic acid and decanoic acid induced a decrease in the twitch force of human ventricular slice preparations. The mean of three independent preparations was calculated and normalized to the contraction force developed at 0.5 Hz of electric stimulation. **c** Representative recording of a contractile force measurement of a human ventricular trabecula with nonanoic acid. Nonanoic acid induced a decrease in the isometric contraction force of a human ventricular trabecula. Force levels were normalized to the maximum response to isoprenaline (5 × 10^−8^ M) prior to nonanoic acid stimulation. Application of isoprenaline at the end of each experiment served to control trabecula viability. **d** Dose dependence of nonanoic acid-induced decrease in isometric twitch force tested on three ventricular trabeculae preparations. *Each point* represents the mean decrease in contractile amplitude measured as percentage of the response to isoprenaline (5 × 10^−8^ M) prior to nonanoic acid stimulation. *Error bars* represent the SEM

To confirm the data obtained from stem cell-derived cardiomyocytes, we measured the effect of OR51E1 ligands on human cardiac tissue ex vivo. A total of 62 measurements were performed using nine different myocardial samples after periods of 1–8 days of slice cultivation. The mean developed force was 1.6 mN among all slices. Five measurements were excluded because of pronounced reactions to DMSO. Slices derived from any of the issue specimen were found suitable for the study, regardless of the etiology of heart failure and the duration of tissue culture. The investigated fatty acids showed a clear structure–activity relationship of their efficacy and kinetics of action. These results are in accord with the receptive field of heterologously expressed OR51E1. Small-molecule fatty acids (C < 9) typically induced a transient decrease in contractility that recovered by up to 60% over the 4-min course of drug application. Larger molecules (C > 9) provoked a stable impairment of contractility down to 33% of the baseline force (Fig. 6a, b; Table S2).

Additionally, we confirmed the negative inotropic effect of OR51E1 agonists observed in heart slices on trabeculae from failing ventricular human myocardium. We found a dose-dependent reduction in the isometric twitch force of isoproterenol stimulated trabeculae under the influence of nonanoic acid; the force was similar to that observed in heart slices and stem cell-derived cardiomyocytes (Fig. 6c, d).

### Possible OR51E1 agonists are present in human epicardial tissue and are elevated in plasma of diabetic patients

To identify the endogenous ligand for OR51E1, we investigated the fatty acid profiles and concentrations in human plasma and epicardial adipose tissue. High-resolution gas chromatographic analysis detected the free fatty acid (FFA) OR51E1 agonists dodecanoic acid (C12:0) and tetradecanoic acid (C14:0) in the plasma of normal controls at low concentrations. Notably, all medium-chain (C:9–C:13) free fatty acid concentrations showed an approximately twofold increase in diabetic patients compared with non-diabetic controls. (Table 1a). However, plasma free fatty acid concentrations may not reflect the situation in epicardial adipose tissue. Therefore, we determined the concentration of the free fatty acids in total epicardial adipose tissue (Table 1b). We found approximately 2, 4, 11 and 23 µmol/l for C9:0, C10:0, C11:0 and C12:0, respectively, for non-diabetic subjects. Since FFAs are mainly present in the aqueous fraction of adipose tissue and since adipose tissue contains approximately 10% water, the interstitial FFA concentrations are about ten times higher, thus in receptor-activating quantities, whereas the identified receptor antagonist was not detected. The fatty acid profile in triacylglycerides in epicardial tissue also demonstrated the presence of OR51E1-activating fatty acids with, however, no difference between diabetic and non-diabetic subjects (Table 1c). Our findings provide the first hints of an involvement of OR51E1 in the regulation of cardiac function by circulating OR51E1-activating FFA and/or by FFA released from epicardial adipose tissue.

**Table 1:** Composition and concentration of fatty acids in plasma and epicardial adipose tissue

Fatty acid	Control	Diabetic	*p* value
(A) Plasma free fatty acid concentration (μmol/l) in controls (*n* = 19) and diabetic patients (*n* = 27)			
Nonanoic acid (C9:0)	0.06 ± 0.03	0.16 ± 0.06	<0.0001
Decanoic acid (C10:0)	0.93 ± 0.88	2.3 ± 1.2	<0.0001
Undecanoic acid (C11:0)	0.20 ± 0.15	0.4 ± 0.15	<0.0001
Dodecanoic acid (C12:0)	3.6 ± 1.8	6.3 ± 2.5	<0.0001
Tridecanoic acid (C13:0)	0.53 ± 0.35	0.97 ± 0.63	0.0151
Tetradecanoic acid (C14:0)	12.2 ± 4.2	14.9 ± 8.8	0.3408
Pentadecanoic acid (C15:0)	1.9 ± 0.62	2.7 ± 1.9	0.1268
Palmitic acid (C16:0)	110 ± 29.9	123 ± 58.4	0.7364
Stearic acid (C18:0)	50.4 ± 10.5	59.4 ± 23.3	0.9071
Oleic acid (C18:1N9)	164 ± 51.8	166 ± 109	0.3403

Fatty acid	Non-diabetic	Diabetic	*p* value
(B) Epicardial adipose tissue free fatty acid concentration (nmol/g tissue wet weight) in controls (*n* = 12) and diabetic patients (*n* = 15)			
Nonanoic acid (C9:0)	18.2 ± 8.3	27.7 ± 15.9	0.0736
Decanoic acid (C10:0)	40.8 ± 45	37.1 ± 57.8	0.8524
Undecanoic acid (C11:0)	106 ± 27	105 ± 59.3	0.9738
Dodecanoic acid (C12:0)	214 ± 215	160 ± 228	0.5389
Tridecanoic acid (C13:0)	7.4 ± 4.5	7.2 ± 3.9	0.9363
Tetradecanoic acid (C14:0)	381 ± 290	225 ± 236	0.1376
Pentadecanoic acid (C15:0)	90 ± 120	23.5 ± 16	0.0426
Palmitic acid (C16:0)	1716 ± 760	1155 ± 760	0.0680
Stearic acid (C18:0)	566 ± 140	516 ± 174	0.4239
Oleic acid (C18:1N9)	1948 ± 914	1285 ± 899	0.0701

Fatty acid	Non-diabetic	Diabetic	*p* value
(C) Fatty acid profile of triacylglycerides of epicardial adipose tissue in non-diabetic (*n* = 15) and diabetic patients (*n* = 14). Fatty acid composition is expressed as percentage of all fatty acids shown			
Nonanoic acid (C9:0)	0.009 ± 0.003	0.01 ± 0.009	0.4941
Decanoic acid (C10:0)	0.15 ± 0.06	0.17 ± 0.08	0.5801
Undecanoic acid (C11:0)	0.15 ± 0.04	0.2 ± 0.11	0.6464
Dodecanoic acid (C12:0)	1.1 ± 0.37	1.3 ± 0.37	0.2278
Tridecanoic acid (C13:0)	0.03 ± 0.01	0.03 ± 0.006	0.7175
Tetradecanoic acid (C14:0)	4.8 ± 1.0	4.9 ± 0.96	0.6269
Pentadecanoic acid (C15:0)	0.38 ± 0.13	0.43 ± 0.08	0.1491
Palmitic acid (C16:0)	28.9 ± 1.8	29.1 ± 2.0	0.8250
Stearic acid (C18:0)	7.2 ± 1.9	6.4 ± 1.6	0.2935
Oleic acid (C18:1N9)	57.3 ± 3.3	57.5 ± 2.5	0.8490

Data were presented as mean ± SD. Non-normally distributed data were transformed to the logarithmic scale for statistical analysis. The comparison of mean variables between groups was carried out using means-independent simple *t* test. *p* < 0.05 was considered statistically significant

## Discussion

### Expression of OR51E1 in the human heart

A variety of ORs are expressed in diverse non-olfactory human tissues, including the brain, the lung, the kidney and the prostate [25, 28, 98, 115]. However, the functional role of these ORs has only been investigated in a few tissues [8, 67, 90, 99, 114]. *OR51E1* was primarily identified in the prostate and is overexpressed in prostate cancer [31, 104, 105]. Further studies have demonstrated the expression of OR51E1 in various healthy and pathologically altered tissues, whereas the function still remains unknown [19, 28, 34, 50].

In the human heart, a broad spectrum of ORs was detected at the RNA level, whereas the odorant receptor *OR51E1* was identified as the highest expressed OR by transcriptome analysis [28]. In addition, our transcriptome analysis results showed that *OR51E1* is also the highest expressed OR in the human fetal heart. The prenatal expression of ectopic OR was described for the rodent developing heart, suggesting a potential role during cardiac development, and for avian embryos, indicating an important role in cell recognition during embryogenesis [22, 23, 26]. The occurrence of *OR51E1* mRNA during the early stages of fetal development suggests a possible role in heart development. In this study, we focused on the expression of OR51E1 in the adult human cardiac ventricles and stem cell-derived cardiomyocytes, which we used as a cardiac in vitro model for the functional analysis of the odorant receptor OR51E1 at the cellular and molecular levels. We detected transcripts of *OR51E1* by RT-PCR in human heart tissue and stem cell-derived cardiomyocytes but not in pluripotent stem cells. Additionally, the expression of OR51E1 could also be validated at the protein level in human heart tissue by immunocytochemical analysis and western blot. Compared to prostate tissue, in the human heart tissue we detected beside the monomeric form of OR51E1 also receptor oligomeres. The detection of higher molecular weight receptor dimers and oligomers are features commonly found after SDS-PAGE for ORs and for GPCR in general [6, 17, 66, 100, 107]. The physiological significance of OR oligomerization is still unknown. Interestingly, different oligomerization states can be observed for the same receptor also in other tissues. The OR51E1 paralogous olfactory receptor 51E2 was found as monomer in the prostate and in human pigment cells in a monomeric and dimeric form [33, 67].

### Activation of OR51E1 in cardiomyocytes

We observed that stimulation of cardiomyocytes with OR51E1 agonist leads to inhibition of the spontaneous Ca^2+^ transients. We showed that this negative chronotropic effect is dose-dependent over the micromolar-to-millimolar range. Detailed analysis revealed that regardless of which type of stem cell-derived cardiomyocytes (hESC- and hIPSC-derived) was investigated, the nonanoic acid-induced effect remained unchanged. We tested whether other medium-chain fatty acids (MCFA) also reduce the frequency of Ca^2+^ spikes and found that the OR51E1 agonists decanoic, dodecanoic and tetradecanoic acid elicited similar effects, whereas compounds that were inactive on the heterologously expressed OR51E1 did not affect the Ca^2+^ spike frequency.

We next aimed to investigate the OR51E1-induced signaling cascade. In olfactory receptor neurons, activation of an olfactory receptor leads to an activation of adenylyl cyclase III via the Gα_olf_ protein. AC-III, in turn, is activated via cAMP CNG-channels [3, 45, 65]. The OR signal transduction pathway in non-olfactory tissues appears to vary depending on the tissue. For most non-olfactory tissues, which express ORs, the initiated pathway is still unknown, but the few examples of pathways that have been determined differ from the classical olfactory signaling cascade [8, 13, 89].

Our results suggest that OR51E1 couples predominantly to the stimulatory G protein, probably to the olfactory G protein. Moreover, the inhibitory G protein could also be a putative interaction partner of OR51E1 due to the finding that Gα_i_ protein was weakly co-precipitated with OR51E1 protein. Recently, it was shown that beside Gα_olf_ also Gα_o_ is functionally coupled to ORs in olfactory sensory neurons [84].

Furthermore, we could observe the involvement of the βγ subunit of the G protein in the nonanoic acid-induced effect. The G protein βγ subunits interact with effector molecules, such as phospholipases, adenylyl cyclase and ion channels, in a manner that leads to their activation or inhibition [16]. Therefore, it is not surprising that the βγ subunit is discussed as a drug target, *inter alia*, for preventing heart failure [53, 88]. OR signaling via βγ subunits was previously described in olfactory sensory neurons and was also demonstrated for ectopically expressed OR51E2, a paralog of OR51E1, in prostate cancer cells [82, 96]. Classical cardiac GPCRs such as the muscarinic acetylcholine receptor M2 and adrenergic receptors also act via the βγ subunit in the heart [87]. Interestingly, bitter taste receptor agonists elicit G protein βγ-dependent negative inotropy in the murine heart [29]. Thus, we suggest that OR51E1 activation leads to GIRK channel opening via G_βγ_ subunit. This opening leads to a hyperpolarization of the cell, which counteracts the HCN channel-induced depolarization, resulting in a reduction of the Ca^2+^ spike frequency.

However, we could not deduce whether the interaction between G protein βγ subunits and other effector molecules primarily mediates the negative chronotropic effect or if the Gβγ subunit only enhances the effect without playing a key role.

We also found that OR51E1 agonists evoked negative inotropic effects on explanted heart preparations. The reduction of contractility of adult human myocardium stimulated with MCFA was dose‐dependent and occurred over a similar dose range as the negative chronotropic effect in stem cell-derived cardiomyocytes, possibly indicating the involvement of a similar G protein βγ-mediated signal transduction. It is well recognized that βγ subunits contribute to the depression of contractility in failing myocardium by targeting the β-adrenergic receptor kinase to the membrane-bound receptors, thereby mediating autologous β-receptor desensitization [74, 83, 85]. However, it is unlikely that this mechanism would be responsible for the reduction in contractility provoked by MCFA in our study because these responses developed with fast kinetics and in the absence of exogenous β-receptor stimulation. There are alternative targets by which G protein βγ-subunits reduce contractility of failing myocardium in a manner independently of β-receptor sensitization [52]. G protein βγ-coupled receptors may activate phospholipase C-β, ERK1/2 and PI3-kinase γ isoform [46, 64], the latter of which has been identified as a negative regulator of cardiac contractility [18]. It is, therefore, tempting to speculate that the negative inotropic effect of the G protein βγ-coupled OR51E1 receptor may be mediated by PI3-kinase γ activation. In addition, it should be noted that the measurements were conducted with myocardium of patients with a heart failure. Human healthy heart tissue was not available in this study due to practical and ethical limitations. Therefore, we could not ensure that cardiac diseases influence the nonanoic acid-induced effect.

### Possible role of OR51E1 in the heart

De-orphanization studies on the recombinant OR51E1 revealed nonanoic acid, which has an unpleasant rancid odor, as an activating ligand [1, 80]. In the present study, we could identify, in addition to nonanoic acid, several MCFA as specific OR51E1 activator. The specificity of recombinant OR51E1 for MCFA was determined by testing a verity of structurally related substances that did not activate the receptor. OR51E1-activating MCFA consist of a 4–14 carbon chain and are mainly provided by food digestion and lipolysis in adipose tissue. In contrast to long-chain fatty acids (LCFA), the dietary intake of short- and medium-chain fatty acids are not significantly associated with the risk of coronary heart disease [41]. A number of studies have indicated that fatty acids not only serve as energy sources but can also act as signaling molecules [43, 68].

We determined the fatty acid profile in human plasma and epicardial adipose tissue as a potential storage site for OR51E1-activating FFA. Recent data indicate a possible role of adipose tissue in modulating cardiac function [12, 42, 69]. It was reported that cardiac adipocytes are able to release substances that suppress the contraction of cardiomyocytes by attenuating intracellular Ca^2+^ levels [49]. We detected medium-chain free fatty acids, which may act as OR51E1 agonists in epicardial adipose tissue. We, therefore, hypothesize that, in addition to dietary intake, OR51E1-activating MCFA in plasma may be released from adipocytes. In plasma of diabetic patients medium-chain free fatty acid concentrations were elevated, whereas the fatty acid profile of triacylglyceride in the fat compartment was not different between both groups. Previously, the fatty profile in epicardial adipose tissue has been determined; however, the MCFA content has not been reported in detail [73]. Thus, OR51E1 is one of the few de-orphanized ORs with an odorant that is also present in the human body.

A great number of reports have shown that FFA-sensing GPCRs play important roles in mediating a variety of physiological processes, such as regulation of energy metabolism mediated by the secretion of hormones and by the regulation of the sympathetic nerve systems and taste preferences, and have demonstrated potential as therapeutic targets for various metabolic and inflammatory disorders, including obesity, type 2 diabetes, atherosclerosis, cardiovascular diseases, ulcerative colitis, Crohn’s disease and irritable bowel disease [5, 21, 38, 43, 61, 91, 93, 97]. *OR51E1* is the highest expressed orphan MCFA-sensing receptor according to our transcriptome data (Table S3). The ligand spectrum of the free fatty acid receptors (FFAR) known to date is only partially overlapping with that of OR51E1. Among the FFAR family, FFAR1 (known as GPR40) and FFAR4 (known as GPR120 and O3FAR1) are classified as medium- to long-chain fatty acid-activated receptors (FFAR1: > C12; FFAR4: C14–C18) [9, 40]. FFAR2 and FFAR3 (known as GPR43 and GPR41) respond to short-chain fatty acids that have fewer than five carbon atoms [10]. Nonetheless, a potential role of FFAR4 in the cardioprotective effect of eicosapentaenoic acid was recently described [24]. Only the free fatty acid receptor GPR84 (C9–C14) is activated by nonanoic acid, whereby nonanoic acid displays the weakest ligand [102]. According to our transcriptome analysis and as previously described, all five receptors are not or only very weakly expressed on mRNA level in the human ventricular myocardium [40]. Because the OR51E1 antagonist and knock-down experiments abolished the nonanoic acid-induced effect completely, we propose that the observed action of MCFA on cardiomyocytes is primarily mediated by OR51E1, but the involvement of other FFARs in sensing OR51E1-activating MCFA cannot be fully excluded in an in vivo situation.

Effects of MCFA on cardiac function were previously reviewed by Francois Labarthe and colleagues [47]. The authors suggested that MCFA not only provide a highly efficient source of energy production as contrary to LCFA they enter the cells by free diffusion and are preferentially directed toward oxidation rather than storage. MCFA also may positively modulate cardiac disease progression when considering the heart’s energy status and contractile dysfunction. In animal heart models, MCFA were reported to improve diabetic cardiomyopathy, prevent cardiac hypertrophy and recover metabolism and contractile function after transient ischemia [2, 15, 27, 48, 55, 63]. The role of OR51E1 in MCFA-mediated benefits for cardiac function or disease progression remains elusive but would be interesting to examine in further studies. Moreover, polyunsaturated fatty acids (PUFAs), such as arachidonic and eicosapentaenoic acid, have been shown to affect Ca^2+^ handling of cardiomyocytes in vitro [54, 57, 110]. PUFAs induce negative inotropy and inhibit Ca^2+^ transients, which leads to the consideration of PUFAs as antiarrhythmic agents. Furthermore, arachidonic acid was described to counteract β-adrenergic receptor-induced stimulation and was consequently considered to perform a cardiac protective function [57, 72].

The results of this study indicate at negative inotropic effects of OR51E1 activation in vivo, however, do not allow for straightforward interpretation of a clinical relevance as further preclinical and eventually clinical evaluation are required to qualify OR51E1 as a cardiovascular drug target. Preclinical testing in animal models should be conducted in future studies.

Interestingly, the OR51E1 and OR51E2 mouse orthologs were found to be expressed in the carotid body and the OR51E2 mouse ortholog, namely Olfr78, acts as a hypoxia sensor. A decreasing oxygen level leads to the production of lactate that in turn affects the breathing circulation via Olfr78 [14]. This finding represents an alternative physiological function of an OR by detecting an endogenous ligand. In the human heart, OR51E1 may act as dietary sensor, which may influence the mechanical and electrical heart function and may possibly be involved in the regulation of the energy metabolism.

In conclusion, our data demonstrate a significant progress towards the characterization of the functional role of ORs in the human heart. We could show that activation of OR51E1 by MCFA induces negative inotropy in human explanted heart preparations and leads to negative chronotropy in stem cell-derived cardiomyocytes, which could be reversed by our identified OR51E1 antagonist. Based on our results, we hypothesize that OR51E1 may play a role in the metabolic regulation of cardiac function. The involvement of heart diseases in pathophysiological processes can only be speculated in the absence of data from animal models or patient studies.

## Electronic supplementary material

Below is the link to the electronic supplementary material.

Supplementary material 1 (PDF 638 kb)
