# Supplementary material for: Medium-chain fatty acids modulate myocardial function via a cardiac odorant receptor
Source: Basic Res Cardiol. 2017 Jan 23;112(2):13. doi: 10.1007/s00395-017-0600-y (PMC5258789; doi:10.1007/s00395-017-0600-y)
Supplement: Supplementary file 1 — Supplementary material 1 (PDF 638 kb) [file 395_2017_600_MOESM1_ESM.pdf]

Supplementary material

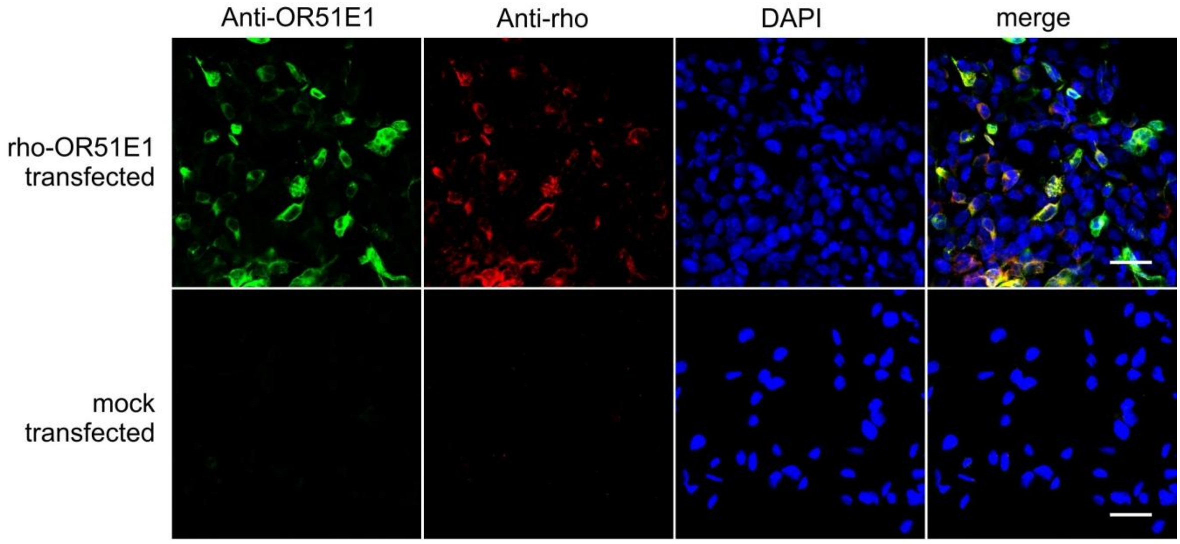

Figure S1. Co-immunocytochemical staining of Hana3A cells transiently expressing OR51E1 with a N-terminal rho-tag, which consists of the first 20 amino acids of rhodopsin. The recombinant rho-OR51E1-protein was detected using an antibody against OR51E1 (green) and an antibody against the n-terminal rho-tag (red). Confocal micrographs of the OR51E1 staining in OR51E1-expressing Hana3A cells are shown. Mock transfected Hana3A cells served as a negative control. Cell nuclei were stained with 4’,6-diamidino-2-phenylindole (DAPI). Co-labeling (yellow) of OR51E1-expressing Hana3A cells indicates specificity of the used anti-OR51E1 antibody. Bar indicates 50 µm.

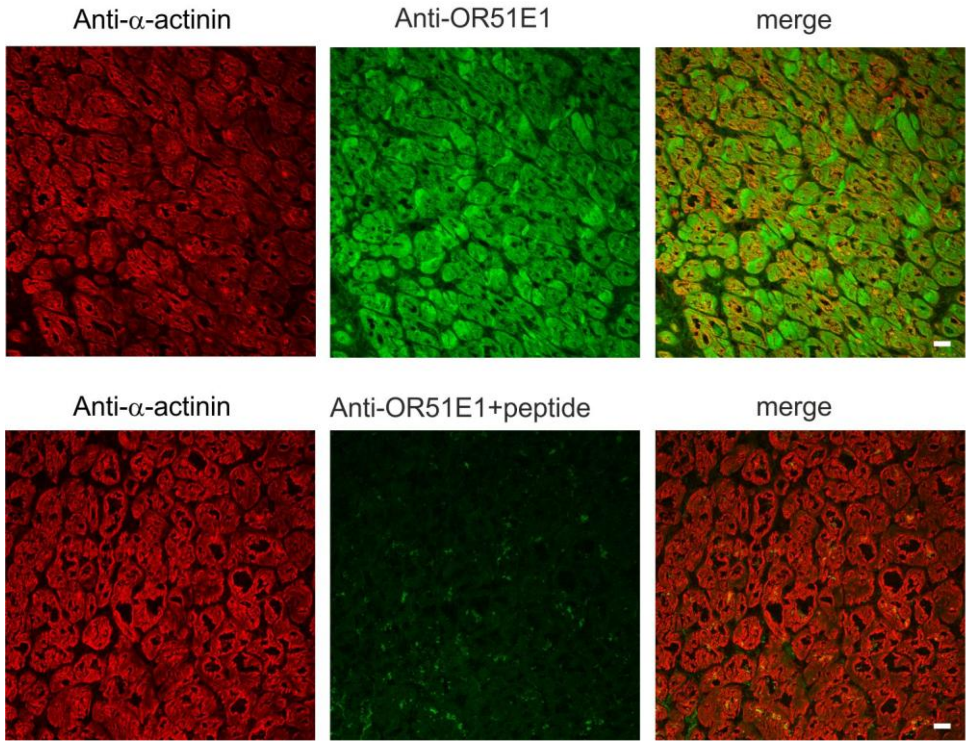

Figure S2. Detection of OR51E1 protein in ventricular myocytes by immunohistochemical staining. Shown are confocal micrographs of OR51E1 immunostaining obtained using an OR51E1 specific antibody in transversal cryosections of human left ventricle. Cardiomyocytes were identified by co-staining with an α-actinin-detecting antibody. Specifity of OR51E1-antibody labeling was controlled by blocking with immunizing OR51E1 peptide (lower panel). Bar indicates 15 µm.

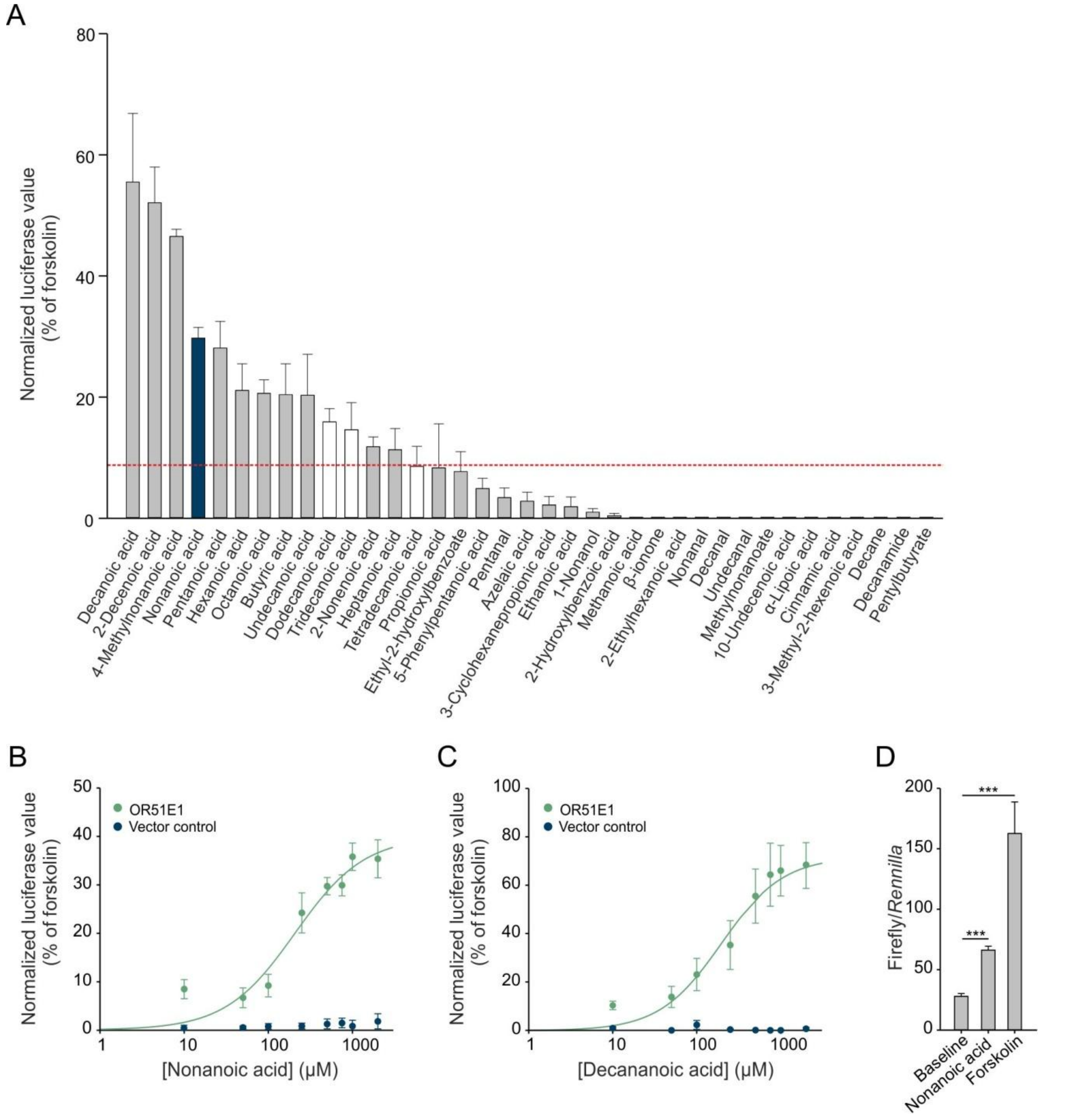

Figure S3. Ligand spectrum of OR51E1. A Structurally related molecules were tested in luciferase reporter assay for their ability to activate heterologously expressed OR51E1, using nonanoic acid as a template (blue bar). An odorant concentration of 500 µM was used (exception: dodecanoic acid [250 µM], tridecanoic acid [250 µM], tetradecanoic acid [100 µM]; because higher concentrations of odorant were not soluble; white bar). Red dashed line represents the response threshold. Bars represent the means of three experiments. Error bars represent the SEM. **B&C** Concentration-response relationships of nonanoic acid (B) and decanoic acid (C) as measured by Luciferase Reporter assays of Hana3A cells heterologously expressing OR51E1. Cellular activation was averaged from 4 biological replicates and normalized to the positive stimulus forskolin, error bars represent the SEM. Regression based on a 4-parameter Hill-Fit. Mock-transfected cells (Vector control) were stimulated to exclude unspecific responses. **D** Unnormalized data measured by Luciferase Reporter assays of Hana3A cells heterologously expressing OR51E1 stimulated with nonanoic acid (200 µM), forskolin and unsimulated cells to demonstrate the baseline value. Bars represent the ratio of firefly luminescence values and *Renilla* luminescence values. The data are shown as the means ± SEM (n=3 independent transfections). Significance was calculated by Student's t-test (*** $p<0.001$).

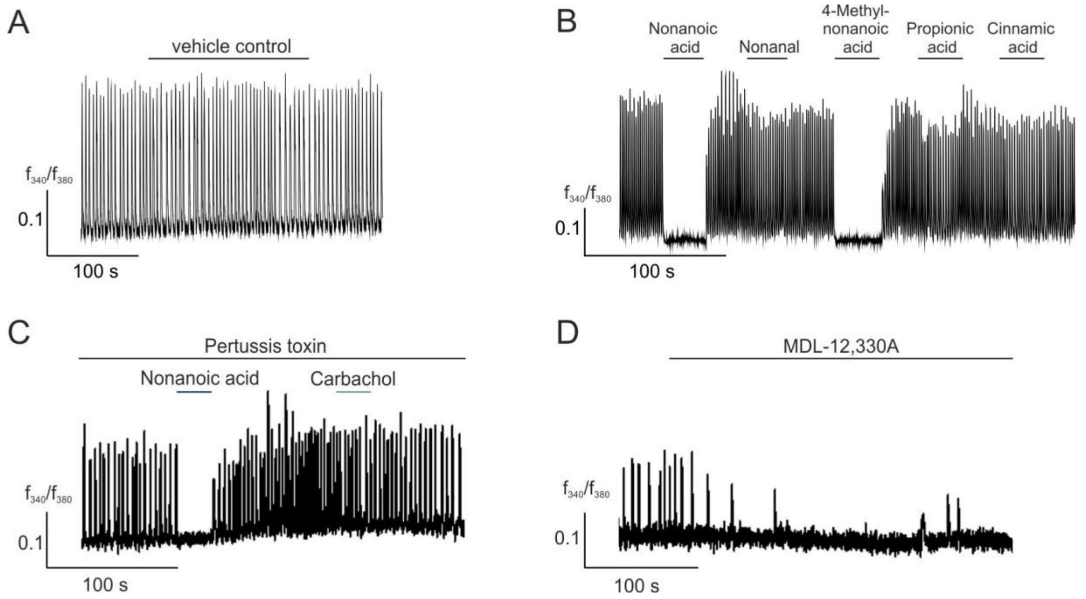

Figure S4. Representative Ca^{2+} imaging traces. A The Ca^{2+} imaging trace of stem cell-derived cardiomyocytes is representative for the vehicle controls (0.1 % DMSO). Application of the solvent did not result in any changes in cytosolic Ca^{2+}. Cytosolic Ca^{2+} levels were monitored as the integrated f_{340}/f_{380} fluorescence ratio expressed as a function of time. **B** Representative Ca^{2+} imaging trace of exemplary selected odorants (500 µM) that activate heterologously expressed OR51E1 (nonanoic acid and 4-methylnonanoic acid) and odorants that are inactive (nonanal, cinnamic acid and propionic acid). The receptive field of heterologously expressed OR51E1 was in accord with the effects on stem cell-derived cardiomyocytes. Cytosolic Ca^{2+} levels were monitored as the integrated f_{340}/f_{380} fluorescence ratio expressed as a function of time. **C** Representative Ca^{2+} imaging traces of stem cell-derived cardiomyocytes treated with G_i protein inhibitor pertussis toxin. After pre-incubation (3 h) with pertissis toxin (1 µg/ml), the carbachol (10 µM)-induced reduction in Ca^{2+} transients was abolished, but the nonanoic acid (500 µM)-induced reduction remained unaffected (left panel). **D** Representative Ca^{2+} imaging traces of stem cell-derived cardiomyocytes treated with an inhibitor that affects spontaneous Ca^{2+} transients (adenylyl cyclase inhibitor MDL-12,330A, 20 µM).

Table S1. Odorant library

Name	Manufacturer	Name	Manufacturer
10-Undecenoic acid	Symrise	Dodecanoic acid	Henkel AG
1-Nonanol	Symrise	Ethanoic acid	Henkel AG
2-Decenoic acid	Henkel AG	Ethyl-2-hydroxybenzoate	Sigma-Aldrich
2-Ethylhexanoic acid	Symrise	Heptanoic acid	Henkel AG
2-Hydroxybenzoic acid	Symrise	Hexanoic acid	Sigma-Aldrich
2-Nonenoic acid	Symrise	Methanoic acid	Symrise
3-Cyclohexanepropionic acid	Symrise	Methylnonanoate	Symrise
3-Methyl-2-hexenoic acid	Symrise	Nonanal	Symrise
4-Methylnonanoic acid	Sigma-Aldrich	Nonanoic acid	Sigma-Aldrich
5-Phenylpentanoic acid	Sigma-Aldrich	Octanoic acid	Sigma-Aldrich
α-Lipoic acid	Symrise	Pentanal	Henkel AG
β-Ionone	Symrise	Pentanoic acid	Sigma-Aldrich
Azelaic acid	Symrise	Pentylbuturate	Symrise
Butyric acid	Sigma-Aldrich	Propionic acid	Sigma-Aldrich
Cinnamic acid	Symrise	Tetradecanoic acid	Sigma-Aldrich
Decane	Sigma-Aldrich	Tridecanoic acid	Henkel AG
Decanal	Sigma-Aldrich	Undecanal	Symrise
Decanamide	Symrise	Undecanoic acid	Sigma-Aldrich
Decanoic acid	Symrise/ Sigma-Aldrich		

Table S2. Structure activity relationship for the negative inotropic effect of selected odorants in slice preparations of explanted human ventricles.

Application of DMSO (vehicle control) did not result in significant changes of isometric contraction. Stimulation with odorants (1 mM) that activate heterologously expressed OR51E1 rapidly reduced twitch amplitude. The effect of short fatty acids ($C \leq 7$) declined within the 4 min of application. The effectivity of highly potent odorants could be demonstrated also at 100 µM concentrations. Odorants that did not activate heterologously expressed OR51E1 or the constitutive receptors of stem cell-derived cardiomyocytes also did not affect adult human myocardium. The mean from two to four independent preparations was calculated and normalized to the contraction force developed at 0.5 Hz electrical stimulation.

	Change in twitch force [%]			
Substance	at 1 mM			at 0.1 mM
	activity	minimum	4 min	minimum
DMSO (0.1 %)	control	-1.8	-1.2	
Nonanoic acid	stable	-58.5	-58.5	-11.2
2-Nonenoic acid	stable	-53.0	-53.0	-11.9
4-Methylnonanoic acid	stable	-67.4	-67.4	-23.7
Decanoic acid	stable	-54.6	-54.6	-6.3
Undecanoic acid	stable	-31.5	-31.5	none
Heptanoic acid	transient	-64.8	-46.6	-34.4
Hexanoic acid	transient	-44.0	-13.6	none
3-Methyl-2-hexenoic acid	inactive	none	none	none
Cinnamic acid	inactive	none	none	none

Table S3. Summary of fatty acid-sensing receptors expressed in human heart

Free fatty acid receptor	adult FPKM	fetal FPKM	Ligand	Reference
**OR51E1**	1.50	1.35	C4:0-C14:0	present study; Saito *et al.*, 2009; Adipetro *et al.*, 2012
GPR84	0.06	0.03	C9:0-C14:0	Wang *et al.*, 2006
FFAR1	0.00	0.00	>C12:0	Briscoe *et a*l., 2006; Itoh *et al.*, 2005
FFAR2	0.08	0.38	<C5:0	Brown *et al.*, 2003
FFAR3	0.06	0.07	<C5:0	Brown *et al.*, 2003
FFAR4 (O3FAR1)	0.07	0.25	C14:0-C18:0	Hirasawa *et a*l., 2004
